# Placenta mesenchymal stem cell-derived extracellular vesicles alleviate liver fibrosis by inactivating hepatic stellate cells through a miR-378c/SKP2 axis

**DOI:** 10.1186/s41232-023-00297-z

**Published:** 2023-10-05

**Authors:** Wenjie Zheng, Saiyan Bian, Shi Qiu, Colin E. Bishop, Meimei Wan, Nuo Xu, Xieyin Sun, Russel Clive Sequeira, Anthony Atala, Zhifeng Gu, Weixin Zhao

**Affiliations:** 1 Research Center of Clinical Medicine, Affiliated Hospital of Nantong University, Medical School of Nantong University, Nantong, 226001 China; 2 Wake Forest Institute for Regenerative Medicine, Wake Forest University Health Sciences, Medical Center Blvd, Winston-Salem, NC 27157 USA

**Keywords:** Mesenchymal stem/stromal cells, Extracellular vesicles, Multicellular organoids, Liver fibrosis, miR-378c

## Abstract

**Background:**

Extracellular vesicles derived from mesenchymal stem/stromal cells (MSCs) have shown therapeutic effects on liver fibrosis. This study aimed to evaluate the effects of extracellular vesicles from placenta-derived MSCs (Pd-MSCs-EVs) on liver fibrosis at 3D/2D levels and explore the potential mechanisms.

**Methods:**

The multicellular liver organoids, consisting of hepatocytes, hepatic stellate cells (HSCs), Kupffer cells, and liver sinusoidal endothelial cells, were observed for growth status, morphological changes, and metabolism. Human transformation growth factor- beta 1 (TGF-β1) was used to induce fibrosis at optimal concentration. The anti-fibrosis effects of Pd-MSCs-EVs were evaluated in liver organoids and HSCs models. Anti-fibrotic content of Pd-MSCs-EVs was identified by multiple experimental validations.

**Results:**

TGF-β1 induced fibrosis in liver organoids, while Pd-MSCs-EVs significantly alleviated fibrotic phenotypes. Following serial verifications, miR-378c was identified as a potential key anti-fibrosis content. In contrast, miR-378c depletion decreased the anti-fibrotic effects of Pd-MSCs-EVs. Additionally, Pd-MSCs-EVs administration repressed TGF-β1-mediated HSCs activation at 2D or 3D levels. Mechanistically, exosomal miR-378c inactivated HSCs by inhibiting epithelial-mesenchymal transition (EMT) through stabilizing E-cadherin via targeting its E3 ubiquitin ligase S-Phase Kinase Associated Protein 2 (SKP2).

**Conclusion:**

Pd-MSCs-EVs ameliorated TGF-β1-induced fibrosis by deactivating HSCs in a miR-378c/SKP2-dependent manner, which may be an efficient therapeutic candidate for liver fibrosis.

**Supplementary Information:**

The online version contains supplementary material available at 10.1186/s41232-023-00297-z.

## Introduction

Liver fibrosis is a complex inflammatory process that results from long-term liver injury, followed by the risk of progressing to liver cirrhosis and even hepatocellular carcinoma (HCC) [1]. Various etiological factors might lead to hepatic fibrosis, including hepatitis virus infection, excessive alcohol consumption, autoimmunity disorders, toxins, obesity, steatosis, and cholestasis [2, 3]. In the past two decades, numerous compounds have shown promising anti-fibrosis functions [4, 5]. However, none of them have been thoroughly validated as an effective therapy in the clinic. Thus, some newer, more clinically applicable approaches have emerged as strong candidates for anti-fibrotic treatment, including gene therapy, regenerative medicine, and nano-therapy [6–8].

Mesenchymal stem and stromal cells (MSCs), defined as fibroblast-like non-hematopoietic cells, have been discovered in various organs and named after their site of origin. Bone marrow MSCs (BM-MSCs), adipose-derived MSCs (Ad-MSCs), umbilical cord-derived MSCs (UC-MSCs), placenta-derived MSCs (Pd-MSCs), dental pulp-derived MSCs (DP-MSCs), and tumor-derived MSCs (Td-MSCs) have been explored in past decades. MSCs have the capacity to differentiate into multiple lineages and undergo osteogenesis, chondrogenesis, and adipogenesis. Notably, they also display significant properties in immunosuppression, anti-inflammation, and anti-fibrosis. Based on their promising therapeutic functions, the effects of MSCs have been evaluated in myocardial infarction, liver cirrhosis, diabetes, and lung injury [9]. Despite considerable benefits, the administration of MSCs may have some drawbacks due in part to biological instability, regulatory limitation, and tumorigenesis risk, which would limit their clinical application in patient-oriented care [10].

Indeed, extensive evidence suggests that most MSC-mediated therapeutic effects might be recapitulated by cell-free factors rather than by direct cell–cell interaction, through the release of various cytokines, chemokines, and extracellular vesicles (EVs) [11]. Among these components, EVs, responsible for the communication between various cell types, are implicated in physiology and pathology. EVs are currently sub-divided into exosomes, microvesicles, and apoptotic bodies according to their size. According to accumulated in vitro and in vivo evidence, MSC-EVs showed massive therapeutic potential for various diseases [12]. Most notably, MSC-derived EVs were recently evaluated in clinical trials, including graft-versus-host disease (GvHD) and chronic kidney disease (CKD) [13, 14]. Although there is no clinical data as of yet, supporting the use of MSCs-EVs in alleviating liver diseases, clear and obvious advantages have been elucidated in animal models of acute liver injury and liver cirrhosis [15].

Liver fibrosis is characterized by widespread scar formation with architectural and functional alterations, mainly contributed by hepatic stellate cell (HSC)-induced accumulation of extracellular cell matrix (ECM) proteins [16]. Given this well-established mechanism, a number of studies attempted to evaluate fibrosis via monolayer culture of HSCs in vitro. However, traditional 2D culture lacks the interactions among several cell types, soluble mediators, the ECM production, and intracellular signaling during hepatic fibrogenesis [17]. Therefore, reliable in vitro models are critical for investigating mechanisms and therapeutic targets of hepatic fibrosis. According to recent study findings, organoid culture systems have shown incredible potential to recapitulate the original organ’s physiology and pathology in vitro [18–20]. While the individual methods may vary, liver organoid models have been applied to the study of hepatocarcinogenesis, screening of anti-fibrosis drugs, and hepato-biliary organogenesis [21, 22]. We have previously constructed a liver organoid model for drug screening and integrated analyses [23, 24]. In the current study, we further developed liver fibrosis models by using a multicellular organoid model and HSC-spheroid model. Based on that model, we comprehensively evaluated the therapeutic effects and potential mechanisms of Pd-MSC-derived extracellular vesicles on hepatic fibrosis at 2D/3D levels.

## Methods

### Cell culture

Placenta-derived MSCs (passage 3) were provided by Wake Forest Regenerative Medicine Clinical Center (RMCC, NC, USA). Pd-MSCs were cultured in α-modified Eagle’s medium (α-MEM, HyClone, MA, USA), supplemented with 15% FBS (HyClone, MA, USA), 17% AmnioMax C-100 Basal Medium (Thermo Fisher Scientific, NY, USA), 2% AmnioMax C-100 supplement (Thermo Fisher Scientific), 1% GlutaMax Supplement (Thermo Fisher Scientific), and 1% Gentamicin (Thermo Fisher Scientific). Human hepatic stellate cells were cultured in Dulbecco’s low glucose modified Eagle’s medium with 2% FBS and 1% Penicillin Streptomycin solution (HyClone, MA, USA). The processes of Pd-MSCs obtainment in RMCC complied with the Declaration of Helsinki. The mimics and inhibitors of miR-378c were purchased from RiboBio (Guangzhou, China) and transfected into cells with RNAiMAX transfection reagent according to the Invitrogen (CA, USA).

### MSC characterization

The adipogenic or chondrogenic differentiation was detected using the StemPro® Adipogenesis or Chondrogenesis Differentiation Kit (Life Science, NY, USA) according to the manufacturer’s introductions, respectively. The osteogenic differentiation was evaluated using the OsteoMAX-XF™ Differentiation Kit (Sigma-Aldrich, MO, USA) according to the manufacturer’s introductions. Following the incubation for indicating time, Oil Red O, alizarin red, and Alcian blue staining (Sigma-Aldrich, MO, USA) were performed to characterize adipogenic, osteogenic, and chondrogenic differentiation, respectively.

MSCs surface markers CD44, CD105, CD73, CD90, CD34, CD45, and CD19 (BD Biosciences, MD, USA) were examined by flow cytometry according to the manufacturer’s introductions. Specific isotype controls were included in this study. In brief, after trypsinization, cells were washed twice in pre-cold phosphate-buffered saline (PBS, HyClone, MA, USA) and suspended in 100μL PBS. Following that, the cells were incubated with antibody solutions as listed above at 4 °C in the dark for 30 min. The samples were washed with PBS, followed by detection using Accuri C6 flow cytometry (BD Biosciences, MD, USA).

### Construction of liver organoids and HSCs-derived spheroids

Liver organoids were established as previously described [24, 25]. In brief, primary human hepatocytes, human Kupffer cells, and human hepatic stellate cells were purchased from TRL/Lonza (NC, USA). Liver sinusoidal endothelial cells were purchased from ScienCell (CA, USA). The cryopreserved cells (hepatocytes, Kupffer cells, hepatic stellate cells, and liver sinusoidal endothelial cell) were thawed and mixed in complete hepatocyte growth medium (HCM, Lonza, NC, USA) as previous instruction. A total of 1200 cells/well in 100μL HCM medium were seeded in ultralow adhesion U-bottomed plates (Corning, MA, USA). After aggregation and compaction for 4 days, each well was replaced with half of the fresh medium every 2 days. For the construction of HSC-derived spheroids, 3000 cells/well in 100μL Dulbecco’s low glucose modified Eagle’s medium with 2% FBS and 1% Penicillin Streptomycin solution. After aggregation and compaction for 3 days, each well was replaced with half-fresh medium every 3 days.

### Extraction of extracellular vesicles

Following the confluence of 80%, pd-MSCs were rinsed in PBS and incubated with serum-free medium for 24 h. Then, the conditioned medium was collected and centrifuged at 400 × g for 5 min, followed by 10,000 × g for 30 min to remove cells and debris. Subsequently, after filtration through a 0.44-μm nylon filter, the supernatants were ultra-centrifuged twice at 100,000 × g for 70 min and resuspended in PBS for storage at − 80 °C. The concentration of exosome was detected by Bradford assay (Bio-Rad, CA, USA) according to the manufacturer’s instructions.

### Transmission electron microscopy and nanoparticle characterization

Samples were absorbed onto 200 mesh glow-discharged carbon-coated grids for 2 min and subsequently dried at room temperature. Then, samples were stained with 2% (w/v) uranyl acetate (UrAc) on ice for 5 min and blotted-off for excess UrAc. Subsequently, the grids were observed under a FEI Tecnai BioTwin Transmission Electron Microscope (FEI Company, OR, USA). The qNano analysis (Izon Science, USA) was used to characterize extracellular vesicles diluted in PBS according to the manufacturer’s instructions.

### Hematoxylin–eosin staining

Organoids were collected from plates and washed twice in PBS, then fixed in 4% paraformaldehyde for 1 h at room temperature. Following washing twice in PBS, organoids were embedded in pre-warmed HistoGel (Thermo Fisher, CA, USA). Then, samples were dehydrated with graded ethanol washes followed by xylene, embedded in paraffin, and cut as sections of 5 μM. After deparaffinized, sections were stained by Autostainer (Leica, IL, USA) for H&E Red staining and observed under a microscope (Zeiss, BW, Germany).

### Immunochemical staining

Formalin-fixed and paraffin-embedded sections were rehydrated and then incubated in 3% H_2_O_2_ for 20 min to reduce endogenous peroxidase activity. After antigen retrieval of 10 mM citrate buffer at 100 °C for 30 min, sections were then blocked with bovine serum albumin (BSA) solution for 1 h at room temperature. Next, samples were incubated in a humidified chamber with anti-collagen I primary antibodies (1:50, Abcam, CA, USA) at 4 °C overnight. After washing twice in tris buffered saline tween (TBST), slides were incubated with horseradish peroxidase-conjugated secondary antibodies (1:100, DAKO, Denmark) for 1 h. Then, sides were subsequently treated with DAB (3,3′-diaminobenzidine) solution and counterstained with hematoxylin. Finally, slides were observed on a microscope (Zeiss, BW, Germany).

### Whole mount immunostaining

Organoids were collected from plates, washed twice in PBS, and then fixed in 4% paraformaldehyde for 1 h at room temperature. After that, samples were permeabilized with 0.1% Triton-X (Sigma-Aldrich, MO, USA) in PBS for 20 min. Following washing twice, samples were blocked in Protein Block Solution (DAKO, Denmark) for 2 h. Then, organoids were incubated with primary antibodies overnight at 4 °C. After washing twice in PBS, organoids were incubated with Alexa Fluor 488 or 594 secondary antibodies (Thermo Fisher Scientific, CA, USA) at room temperature for 1 h. Finally, organoids were washed twice in PBS and stained with DAPI prior to fluorescent imaging. At last, samples were imaged under a confocal microscope (Leica, IL, USA). Negative controls were included with the primary antibody replaced by blocking solution.

### Viability assay of organoids/ spheroids

The live/dead assay was performed using LIVE/DEAD® Viability/Cytotoxicity Kit (Thermo Fisher Scientific, CA, USA) according to the manufacturer’s instructions. In brief, the liver organoids or HSCs-derived spheroids were collected from indicated wells and washed in PBS for 3 times. After that, the organoids /or spheroids were stained with calcein AM and ethidium homodimer-1 at a ratio of 1:4 for 30 min at room temperature. The fluorescence of the samples was observed under a confocal microscope (Leica, IL, USA).

### EdU proliferative assay

The proliferative assay was conducted using Click-iT™ Plus EdU Alexa Fluor™ 594 Imaging Kit (Thermo Fisher Scientific, CA, USA) according to the manufacturer’s instructions. HSCs or HSC-derived spheroids were treated with EdU solution and incubated for 24 h at 37 °C. After incubation, samples were incubated in 4% formaldehyde in PBS for 15 min at room temperature. After washing with 3% BSA in PBS, samples were treated with 0.5% Triton X-100 for 20 min at room temperature. Following washed in 3% BSA solution, the samples were incubated in Click-iT® Plus reaction cocktail for 30 min. After being stained with Hoechst 33342 solution, the HSCs or HSC-derived spheroids were visualized using a fluorescence microscope or a confocal microscope (Leica, IL, USA), respectively.

### Proliferation analysis

HSCs were seeded into 96-well plates at a density of 1.5 × 10^3^ cells/ well. Then cell viability was measured at the indicated time points using the Cell Counting Kit 8 (CCK8, Thermo Fisher Scientific, CA, USA) according to the manufacturer’s instructions. The absorbance of each well was measured using a plate reader (SpectraMax M5, Molecular Devices, CA, USA).

### Wound healing and transwell assays

For the wound healing assay, HSCs were seeded into 6-well plate at the density of 1 × 10^6^ cells/ well. After the confluence of 80%, cells were then scratched using sterile tips. After that, cells were treated with extracellular vesicles or control solution for 24 h. The cell migration was evaluated according to the average width of the wounded gaps measured at each time point.

For the transwell assay, HSC cells were seeded into transwell chambers (Sigma-Aldrich, MO, USA) at a density of 5 × 10^4^ cells/well in 200 μL serum-free DMEM medium. Then, 600 μL DMEM containing 20% FBS was added into the lower chamber. After being treated with extracellular vesicles or control solution for 24 h, the migrated cells were fixed with 4% paraformaldehyde and stained with 0.2% crystal violet and then observed under a light microscope (Leica, IL, USA).

### Extracellular vesicle labeling

The extracellular vesicles were labeled with a PKH26/ and PKH67 Fluorescent Cell Linker Kit (Sigma-Aldrich, MO, USA) according to the manufacturer’s protocol. Labeled extracellular vesicles re-suspended in DMEM were added into HSCs or organoids/spheroids and then incubated for 4 h or 24 h, respectively. Cells or organoids/ spheroids were washed in PBS 3 times, fixed in 4% paraformaldehyde for 30 min, and then stained with DAPI for 30 min at RT. Finally, HSCs or organoids/spheroids were observed under a fluorescence microscope or a confocal microscope (Leica, IL, USA), respectively.

### Apoptosis and cell cycle analyses

Apoptosis of HSCs was detected by flow cytometry analysis using eBioscience™ Annexin V Apoptosis Detection Kit (Thermo Fisher Scientific, CA, USA) according to the manufacturer’s protocol. After being treated with extracellular vesicles or control solution for 24 h, the cells were collected and washed three times in pre-cold PBS. The samples were subsequently stained with fluorochrome-conjugated Annexin V for 15 min at room temperature, followed by washing with Binding Buffer. Finally, cells were treated with propidium iodide staining solution and detected in Accuri C6 flow cytometry (BD Biosciences, MD, USA).

Cell cycle analysis was performed using FxCycle™ PI/RNase Staining kit (Thermo Fisher Scientific, CA, USA) according to the manufacturer’s instructions. HSCs treated with exosome or control solution were washed twice with ice-cold PBS and resuspended in pre-cold 70% ethanol. After being washed with PBS and treated with DNase-free Rnase A for 30 min in 37 °C, the samples were incubated for 30 min at room temperature. Results were analyzed by the Modfit software (Verity Software House, CA, USA).

### Enzyme-linked immunosorbent assay

Supernatant of each well was collected to perform enzyme-linked immunosorbent assay (ELISA) according to the manufacturer’s instructions for each kit. Albumin production was measured using Human Albumin ELISA kit (Alpha Diagnostic International, NJ, USA). Inflammatory factors were measured using Human Interleukin-1 β (IL-1β) ELISA kit and Human Tumor Necrosis Factor-α (TNF-α) kit (Sigma-Aldrich, MO, USA). Pro-collagen production was detected using Human Pro-Collagen I alpha 1 ELISA Kit (Abcam, CA, USA). Samples were measured at the wavelength of 450 nm using plate reader (SpectraMax M5, Molecular Devices, CA, USA). Each test was conducted at least three times.

### Viability assay

Viability assay was conducted using CellTiter-Glo® 3D Luminescent Cell Viability Assay Kit (Promega, WI, USA) according to the manufacturer’s introductions. Each well of organoid was treated with 100 μL CellTiter-Glo® 3D Reagent and vigorously mixed. Following incubation at room temperature for an additional 30 min, luminescent signal was read on a plate reader (Molecular Devices, CA, USA). The supernatants were used as the blank control. Each experiment was performed in triplicate on three independent experiments. For mono-culture, CCK-8 assay was conducted to evaluate cell proliferation according to the manufacturer’s protocol. Briefly, cells were placed into a 96-well plate in the density of 3 × 10^3^/well, followed by incubating with CCK-8 reagent (DOJINDO, Kumamoto, Japan) for 2 h. Then, the optical density was detected at the wavelength of 450 nm by using a plate reader.

### Quantitative real-time PCR

The total RNA was extracted by RNeasy Mini kit (Qiagen, CA, USA) according to the manufacturer’s introductions. cDNA was synthesized using High-Capacity cDNA Reverse Transcription Kit (Applied Biosystems, CA, USA) according to the manufacturer’s instructions. Quantitative real-time PCR (qRT-PCR) was performed with PowerUp™ SYBR™ Green Master Mix (Thermo, NY, USA) according to the manufacturer’s instructions in the Applied Biosystems RT-PCR machine (Thermo, NY, USA). GAPDH was chosen as a reference gene. The experiments were conducted in triplicates in three independent experiments. The sequences of primers sequences are shown in Table S1.

### Western blotting

Organoids were harvested and treated with RIPA Lysis Buffer (Sigma-Aldrich, MO, USA) to extract protein. After that, samples diluted with loading buffer were added to the 10% sodium dodecyl sulfonate (SDS) gel. Following the transfer to the polyvinylidene fluoride (PVDF, Bio-Rad, CA, USA) membrane, samples were blocked in a 5% BSA solution. Then, membranes were incubated with primary antibody solution (anti-collagen I, anti-CD63, CD81, CD9, Abcam, USA; anti-α-smooth muscle actin, Sigma, USA; E-cadherin, Vimentin, Ub, proteintech, China) at a ratio of 1:1000 overnight at 4 °C. After washing three times in TBST, samples were incubated in solution of horseradish peroxidase-linked secondary (Thermo, NY, USA) for 2 h at room temperature. Then membranes were washed with TBST for three times, followed by visualizing with Pierce ECL Western Blotting Substrate (Thermo, NY, USA).

### Hepatic satellite cell activation model

TGF-β1 was utilized to activate human hepatic satellite cell LX-2 over time (10 ng/ml for 24 h or 48 h) or concentration (5 ng/ml or 10 ng/ml for 24 h). The mRNA expression of the typical feature genes was assessed using RT-PCR and Western blotting.

### Mouse liver fibrosis model

C57BL/6 mice were divided into two groups: control and intervention. Each group had 20 mice. CCL4 (5 ml/kg) was administered intraperitoneally twice a week to the intervention group, whereas normal saline was administered to the control group. Four weeks later, the liver specimens from each group were collected for Masson, Sirius Red, and immunochemistry analysis. All procedures in this animal study were approved by the Nantong University Animal Care and Use Committee.

### Exosomal bioactive content identification

The total RNA was extracted from organoids treated with TGFβ1 and Pd-MSC-derived extracellular vesicles by RNeasy Mini kit (Qiagen, CA, USA) according to the manufacturer’s introductions. miRNA identification was performed on a BGISEQ-500 platform by Wake Forest University (NC, USA). SOAPnuke software was performed to filter the raw data, and HISAT software was used to map them to the Genome Reference Consortium human Build 38 (GRCh38.p12). DESeq2 was conducted to identify differentially expressed miRNAs (fold change ≥ 2 with *p* < 0.05).

### Cycloheximide(CHX)-based protein stability and ubiquitination assays

HSCs were treated with 20 μg /ml CHX (Sigma, USA) at different times. The cells were then harvested and lysed in preparation for western blotting. Densitometry was used to analyze band intensities semi-quantitatively with Adobe Photoshop 2022. For ubiquitination assay, HSCs were transfected with miR-378c mimics, inhibitors, and/or OE-SKP2 plasmids and then lysed cells for immunoprecipitation and IB with indicated antibodies.

### Statistical analysis

All statistical analyses were performed using SPSS 19.0 (IBM Corporation, CA, USA) and GraphPad Prism 7 software (CA, USA). The data are presented as mean ± standard deviation (SD). Comparisons between groups were conducted using 2-tailed Student’s* t*-test or two-way ANOVA. All assays were conducted at least three times. *P* value < 0.05 was considered statistically significant.

## Results

### Multicellular liver organoid formation

**Fig. 1:**
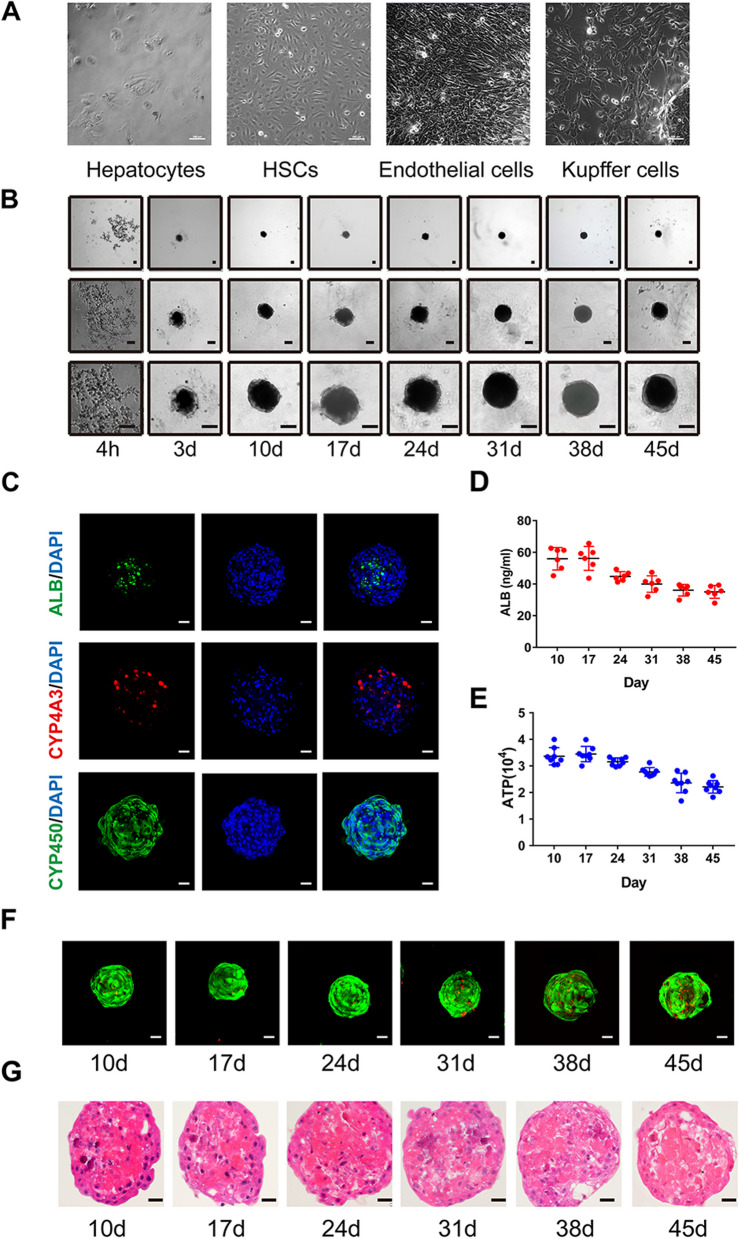
Construction of multicellular liver organoids. **A** Representative images of four types of cells for the construction of liver organoids (hepatocytes, hepatic stellate cells, Kupffer cells, and liver sinusoidal endothelial cells). **B** The morphological observation of liver organoids from 4 h to the 45th day. **C** Determination of liver-specific markers (Albumin, CYP3A4, and CYP450) in liver organoids. **D** Albumin secretion in liver organoids from the 10th day to the 45th day detected by ELISA. **E** The growth status of liver organoids from the 10th day to the 45th day detected by 3D viability assay. **F** Live/dead staining was performed in liver organoids from the 10th day to the 45th day. **G** H&E staining of liver organoids from the 10th day to the 45th day. HSCs, hepatic stellate cells; ALB, albumin. Bar scale, 50 μm

Liver organoids were constructed using 4 types of cells, including hepatocytes, HSCs, Kupffer cells, and liver sinusoidal endothelial cells (Fig. 1A). Then, the morphology changes of the liver organoids were observed from 4 h to the 45th day after seeding (Fig. 1B). While the cells individually maintained their characteristic features initially upon seeding, the four cell types began aggregating by the 3rd day and continued to self-assemble into 3D organoid structures. Aside from enlarging in size, the organoids showed no observable alterations from the 17th day to the 45th day. In order to characterize the liver organoids over time, several liver-specific markers (ALB, CYP4A3, and CYP450) were assessed and visually detected by immunofluorescence staining (Fig. 1C). In addition, the growth status and progression of liver organoids were followed from the 10th day to the 45th day. Liver organoids-secreted ALB was maintained during early weeks and mildly decreased from the 24th day (Fig. 1D). Additionally, the 3D luminescent cell viability assay indicated that ATP values of the organoids temporarily increased at first 2 weeks and then decreased with time (Fig. 1E). Similarly, the Live/Dead assay showed that the liver organoids maintained their structure and viability in the early weeks but slowly dead cells (red fluorescence) accumulated and a decline in viability was observed over time (Fig. 1F). H&E staining was also conducted to evaluate the internal architecture of the organoids at different time points. Although there was no significant change in the overall structure of the liver organoids, less clearly defined nuclear staining was observed at later time points (Fig. 1G). Taken together, these results suggested the multicellular self-assembled liver organoids could maintain their structure and viability in vitro during the initial weeks following seeding but with a decline in these features during extended culture duration.

### Fibrosis induction in liver organoid

**Fig. 2:**
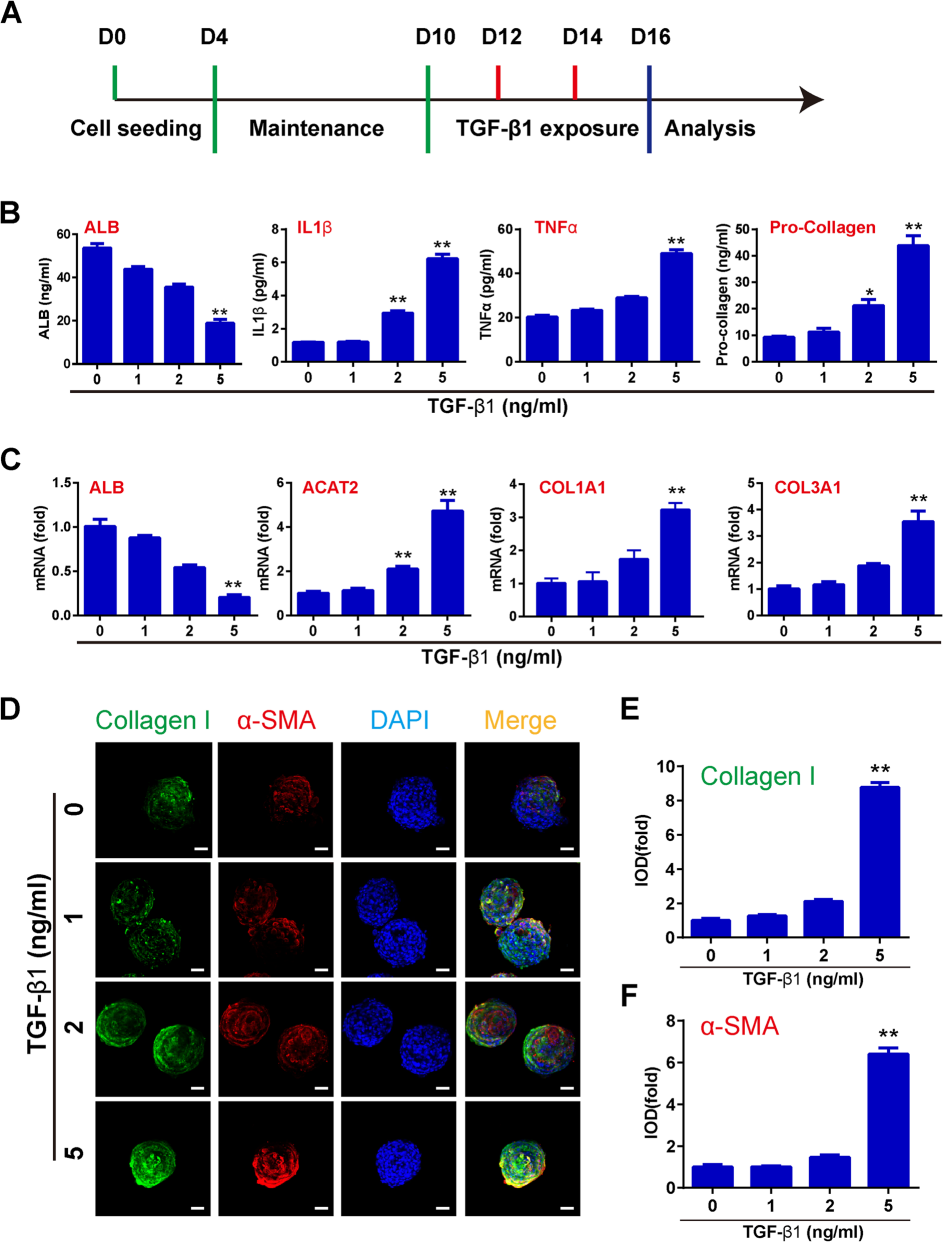
Fibrosis induction in liver organoids by TGF-β1 treatment. **A** The workflow of organoid maintenance and TGF-β1 administration. **B** After the treatment of TGF-β at different concentrations, the secretion of albumin, IL-1β, TNF-α, and Pro-collagen of liver organoids was detected by ELISA. **C** The mRNA expression of ALB, ACAT2, COL1A1, and COL3A1 was detected by RT-qPCR. **D** Immunofluorescence staining of collagen I and α-SMA detected by confocal microscopy. **E** Semi-quantitative analysis of collagen I staining intensity in **D**. **F** Semi-quantitative analysis of α-SMA staining intensity in **D**. TGF-β1, transformation growth factor- beta; ALB, albumin; α-SMA, α-smooth muscle actin. Bar scale, 50 μm. **P < 0.01

Based on the results above, we constructed a fibrosis model of liver organoids utilizing TGF-β1 exposure. As shown in the workflow (Fig. 2A), the organoids were maintained in a regular medium for 10 days, followed by the treatment of TGF-β1 at different concentrations every 2 days. Then, any morphological and molecular alterations were detected on the 16th day. While the growth status and ATP values displayed no apparent change after TGF-β1 exposure (Figure S1A and B), ALB secretion was significantly repressed, while simultaneously the secretion of inflammatory markers (IL-1β and TNF-α) and fibrotic markers (pro-collagen) increased in a dose-dependent manner (Fig. 2B). Consistently, TGF-β1 treatment led to an obvious downregulation and upregulation of ALB expression and fibrotic markers (ACAT2, COL1A1, and COL3A1) in mRNA levels, respectively (Fig. 2C). Furthermore, the TGF-β1-induced enhancement of α-SMA and Collagen I expression was confirmed by immunofluorescence using confocal microscopy (Fig. 2D–F). Taken together, these results strongly indicated that TGF-β1 could induce fibrotic phenotype of liver organoid in dose-dependent manner.

### Characterization of Pd-MSCs derived extracellular vesicles

**Fig. 3:**
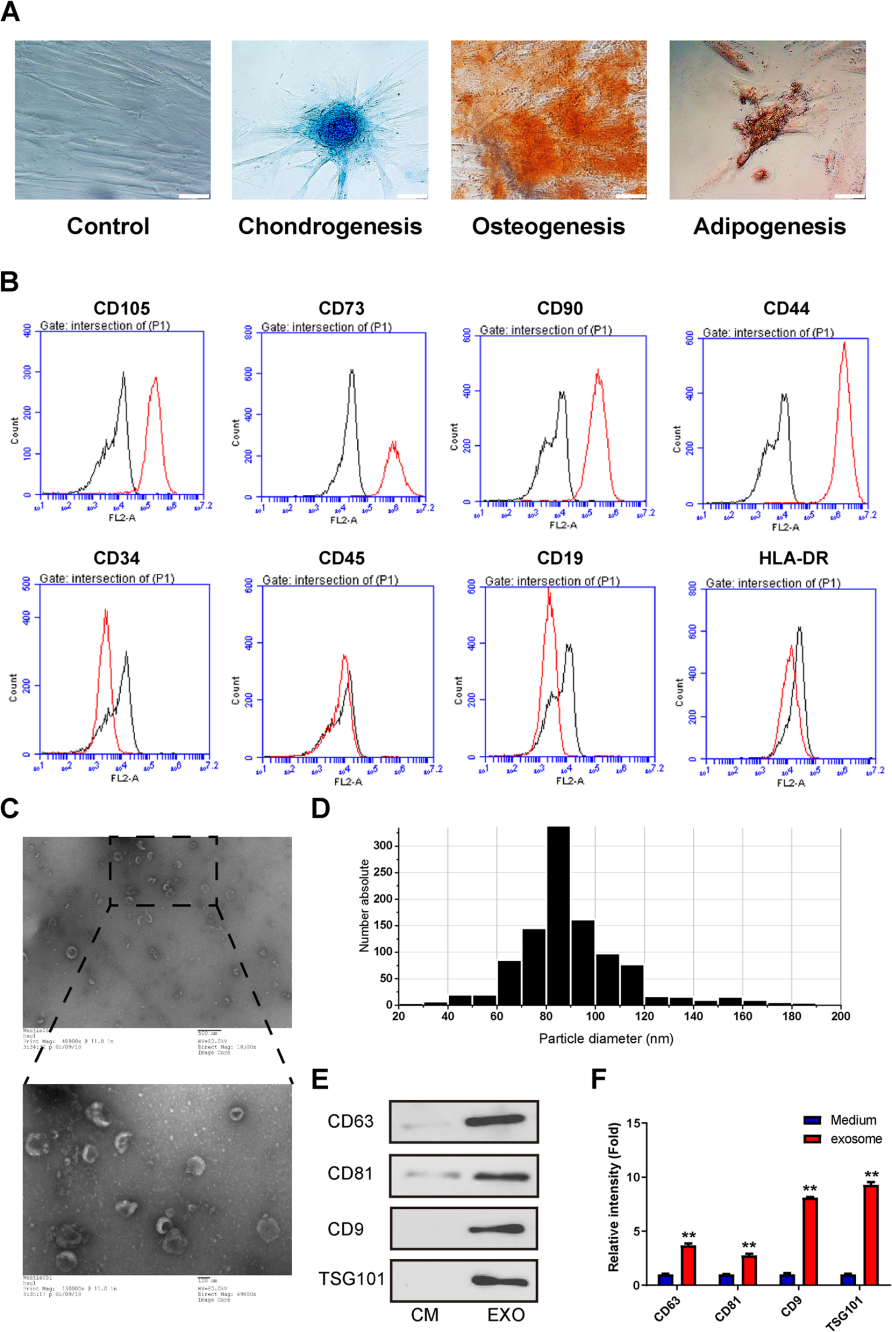
Characterization of Pd-MSCs and their extracellular vesicles. **A** Differentiation of MSCs displaying chondrogenesis, osteogenesis, and adipogenesis, which was validated by alcian blue, alizarin red, and oil red O staining, respectively. **B** Flow cytometric analyses of phenotypic markers of MSCs (positive markers, CD105, CD73, CD44, and CD90; negative markers, CD34, CD45, CD19, and HLA-DR). **C** Morphology of extracellular vesicles observed by transmission electron microscopy. **D** Size distribution of Pd-MSCs-extracellular vesicles measured by qNano analysis. **E** Exosome-specific markers CD9, CD63, CD81, and TSG101 were detected in conditioned medium of Pd-MSCs and exosome solution by western blotting. **F** Semi-quantitative analysis of protein expression in **E**. CM, conditioned medium; EVs, extracellular vesicles. Bar scale, 50 μm. *P < 0.05; **P < 0.01

MSCs were isolated from human placenta and evaluated for capacity to differentiate into multiple lineages, including chondrogenesis, osteogenesis, and adipogenesis (Fig. 3A). Then, FACS analysis demonstrated that Pd-MSCs positively expressed CD105, CD73, CD44, and CD90 and negatively expressed CD34, CD45, CD19, and HLA-DR (Fig. 3B). Extracellular vesicles from conditioned medium were isolated by differential ultracentrifugation. Under transmission electron microscopy, the Pd-MSC-derived extracellular vesicles presented classical structure of round or oval bilayer membrane (Fig. 3C). qNano analysis showed that the diameter of the Pd-MSCs-exosome was approximately 60–120 nm (Fig. 3D). Western blotting showed that extracellular vesicles highly expressed exosome-positive markers CD63, CD81, CD9, and TSG101 (Fig. 3E, F).

### The uptake of Pd-MSCs-EVs by liver organoids

**Fig. 4:**
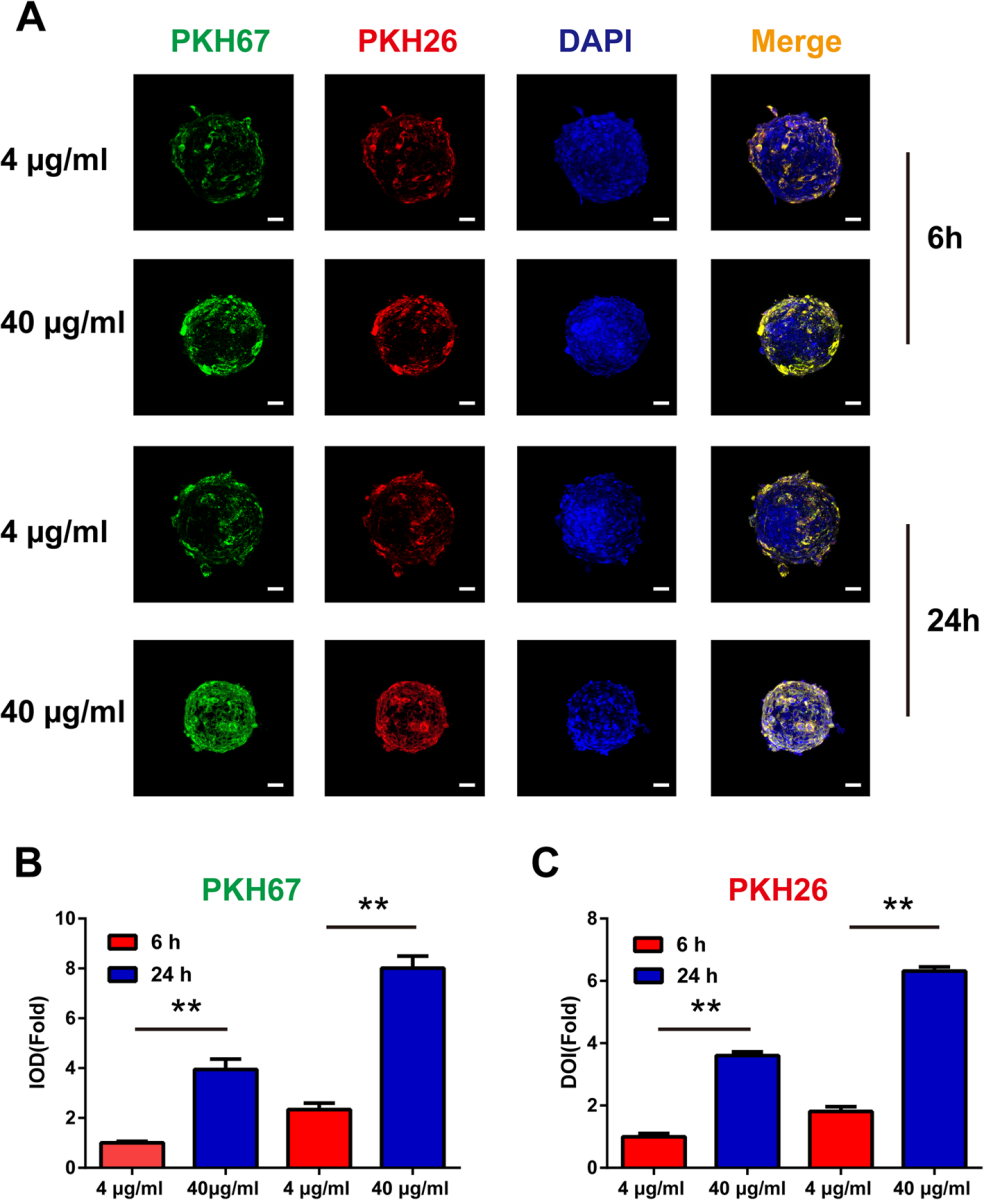
The uptake of Pd-MSCs-EVs by liver organoids. **A** The Pd-MSCs-EVs at different concentrations were simultaneously pre-stained with PKH67 and PKH26 and then administrated into liver organoids. The uptake of extracellular vesicles was observed after 6 h or 24 h. **B** Semi-quantitative analysis of PKH67 staining in **A**. **C** Semi-quantitative analysis of PKH26 staining in **A**. Bar scale, 50 μm. *P < 0.05; **P < 0.01

Given the differences in growth characteristics between 2D cell culture and 3D organoid culture, the uptake of MSCs-EVs at different concentrations was measured at various time points by simultaneous pre-labeling with PKH67 and PKH26. As shown in Fig. 4A, organoids treated with 40 μg/ml extracellular vesicles for 24 h presented with higher fluorescence intensity than groups treated with lower concentrations or treated for shorter time intervals. Accordingly, semi-quantitative analysis demonstrated that administration with 40 μg/ml extracellular vesicles for 24 h led to higher fluorescence intensity of PKH26 and PKH67 in liver organoids, indicating a higher uptake of extracellular vesicles by liver organoids (Fig. 4B, C). Thus, the concentration and exposure time of extracellular vesicles were chosen for further organoid-related experiments.

### Pd-MSCs-EVs alleviated TGF-β1-induced fibrosis in liver organoids

**Fig. 5:**
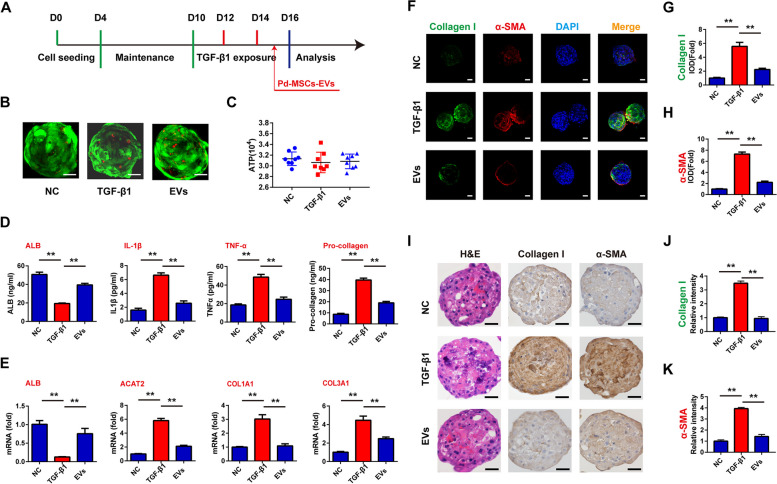
The anti-fibrosis effects of Pd-MSCs-EVs on liver organoids. **A** The workflow of TGF-β1 and Pd-MSCs-EVs administration in liver organoids. **B** Live/dead staining was conducted in liver organoids following TGF-β1 administration. **C** The effects of Pd-MSCs-EVs on the growth status of liver organoids were detected by 3D viability assay. **D** Following the treatment of Pd-MSCs-EVs, the secretion of albumin, IL-1β, TNF-α, and Pro-collagen of liver organoids was detected by ELISA. **E** The mRNA expression of ALB, ACAT2, COL1A1, and COL3A1 was detected by RT-qPCR. **F** Immunofluorescence of collagen I and α-SMA detected by confocal microscopy. **G** semi-quantitative analysis of collagen I staining intensity in **F**. **H** Semi-quantitative analysis of α-SMA staining intensity in **F**. **I** The H&E staining and collagen I and α-SMA immunohistochemical staining of liver organoids treated with TGF-β and Pd-MSCs-EVs. **J** Semi-quantitative analysis of collagen I immunohistochemical staining intensity in I. **K** Semi-quantitative analysis of α-SMA immunohistochemical staining intensity in I. TGF-β1, transformation growth factor- beta; ALB, albumin; α-SMA, α-smooth muscle actin. Bar scale, 50 μm. **P < 0.01

After sustained exposure to TGF-β1, the organoids were treated with 40 μg/mL concentration of extracellular vesicles on the 15th day. Subsequently, the organoids in different groups were simultaneously evaluated on the 16th day (Fig. 5A). As shown in Fig. 5B, C, the administration of Pd-MSCs-EVs had no obvious effects on the growth status and ATP values of TGF-β-treated liver organoids. However, Pd-MSCs-EVs resuscitated albumin secretion and reversed the secretion of inflammatory markers and fibrotic markers induced by TGF-β1 exposure (Fig. 5D). For mRNA levels, extracellular vesicle treatment upregulated ALB expression and downregulated fibrotic markers (ACAT2, COL1A1, and COL3A1), which partially abrogated the TGF-β1 mediated effects on liver organoids (Fig. 5E). Consistently, immunofluorescence analysis showed that administration of Pd-MSCs-EVs significantly repressed the fluorescence intensity of α-SMA and Collagen I upregulated by TGF-β1 (Fig. 5F–H). In addition, immunohistochemical analysis indicated that the staining intensity of α-SMA and Collagen I was significantly decreased after administration of Pd-MSCs-EVs (Fig. 5I–K). The results above suggested that the Pd-MSCs-EVs could inhibit the fibrotic phenotype of liver organoids induced by TGF-β1.

### Pd-MSCs-EVs abated TGF-β1-induced fibrosis by delivering miR-378c

**Fig. 6:**
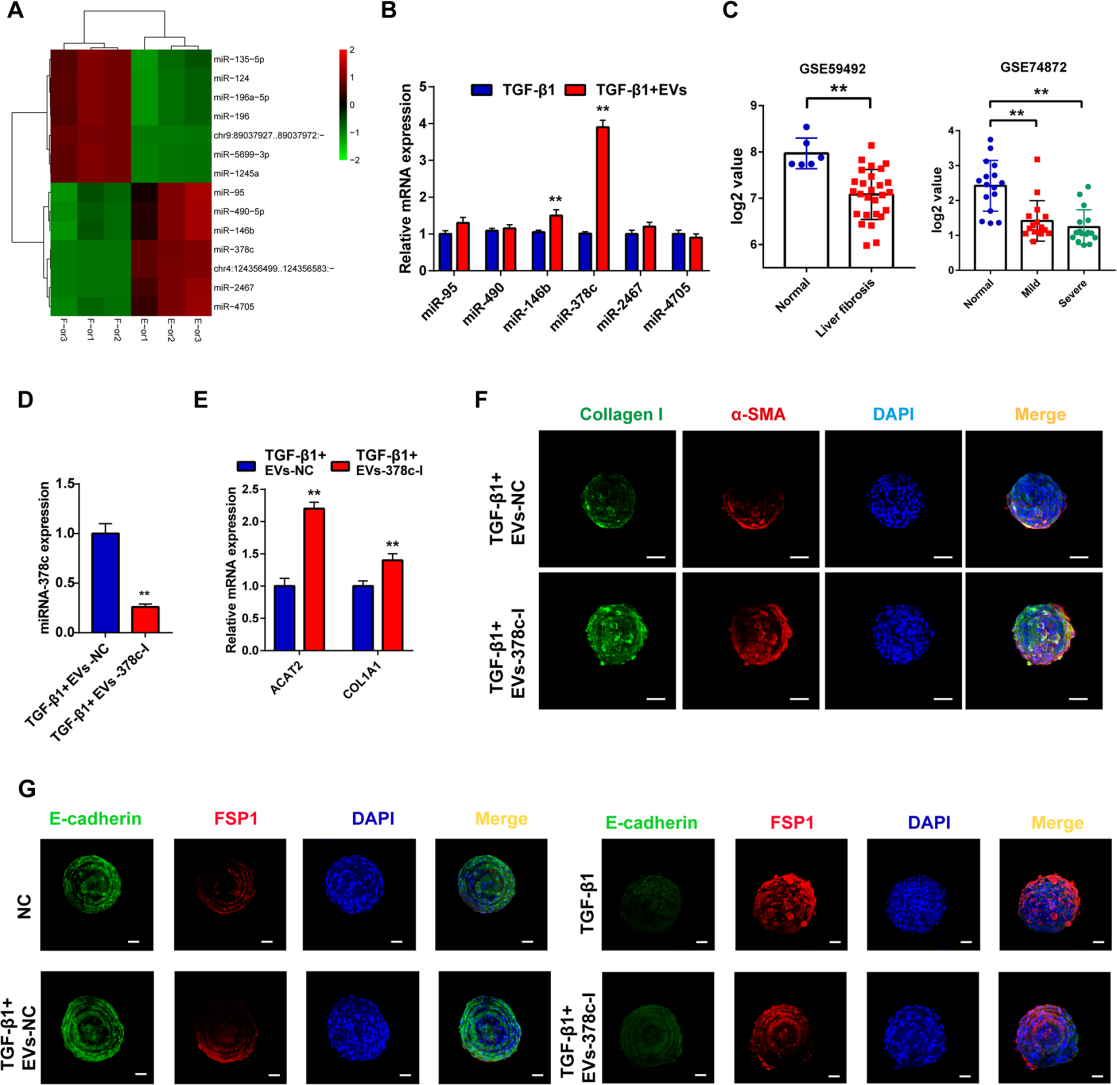
Identifying miR-378c as effective anti-fibrosis content in Pd-MSCs-EVs. **A** miR-seq was performed to identify differential miRNAs between fibrosis organoid model with or without Pd-MSCs-EVs administration. **B** The differential miRNAs were validated by RT-qPCR. **C** miR-378c expression in two GEO datasets regarding liver fibrosis. **D** miR-378c expression in extracellular vesicles derived from MSCs with or without miR-378c inhibitors. **E**, **F** The expression of fibrotic markers in different groups was detected by RT-qPCR and immunofluorescence. **G** The expression of EMT markers in different groups was detected by immunofluorescence. TGF-β1, transformation growth factor- beta; α-SMA, α-smooth muscle actin. **P < 0.01

EV-derived miRNAs may be important contents that mediate the effects of the extracellular vesicles. Thus, we further conducted omics analyses to identify differential miRNAs between fibrotic organoids with or without Pd-MSCs-EVs treatment. As shown in Fig. 6A, 6 known miRNAs were significantly upregulated (logFC > 1, *P* < 0.05) in Pd-MSCs-EVs-treated organoids. Further PCR validation found that miR-378c presented the most obvious difference between the two groups (Fig. 6B). In addition, bioinformatic analysis showed that miR-378c expression was significantly decreased with the initiation of liver fibrosis (Fig. 6C). Extracellular vesicles derived from Pd-MSCs with miR-378c inhibitor treatment (EVs-378c-I) showed less anti-fibrosis effects on TGF-β1-exposed liver organoids (Fig. 6D–F). Previous studies indicated that epithelial-mesenchymal transition (EMT) process was correlated with liver fibrosis. Besides, TGF-β1 was considered to induce EMT in various cell types. Thus, EMT process was evaluated in liver organoids treated with TGF-β1, Pd-MSCs-EVs, and/or EVs-378c-I. As analyzed by confocal microscopy (Fig. 6G), TGF-β1 could increase the expression of FSP1 with the downregulation of E-cadherin. However, Pd-MSCs-EVs reversed the TGF-β1- induced upregulation of these EMT markers. In accordance, EVs-378c-I brought no obvious changes to the EMT phenotypes in TGF-β1-induced organoids. The results above demonstrated that miR-378c might be crucial molecules that are responsible for the anti-fibrosis effects of Pd-MSCs-EVs.

### miR-378c inhibited TGF-β1-activated HSCs

Activated HSCs were considered to play vital roles in the hepatic fibrogenesis through secreting ECM. Then, the effects of Pd-MSCs-EVs were evaluated on 3D HSC-derived spheroids. Consistent with the liver organoids, 40 μg/ml Pd-MSCs-EVs was administrated in HSC-derived spheroids for 24 h. Then, the uptake was confirmed by PKH67 and PKH26 staining (Figure S2A). Live/dead assay showed that TGF-β1 or Pd-MSCs-EVs treatment might have less effect on the survival of HSC-derived spheroids (Figure S2B). However, the proliferation and ATP of HSC-derived spheroids significantly decreased following Pd-MSCs-EVs administration (Figure S2C-E). Remarkably, Pd-MSCs-EVs could inhibit the expression of α-SMA(ACACT2) and Collagen I (COL1A1) of HSCs or their derived spheroids upregulated by TGF-β1 treatment (Figure S3A). Consistently, downregulation of α-SMA and Collagen I staining intensity was also observed in HSC-derived spheroids treated with Pd-MSCs-EVs by immunofluorescence and immunohistochemistry (Figure S3B-D). This evidence demonstrated that Pd-MSCs-EVs could repress TGF-β1-induced activation of HSCs.

**Fig. 7:**
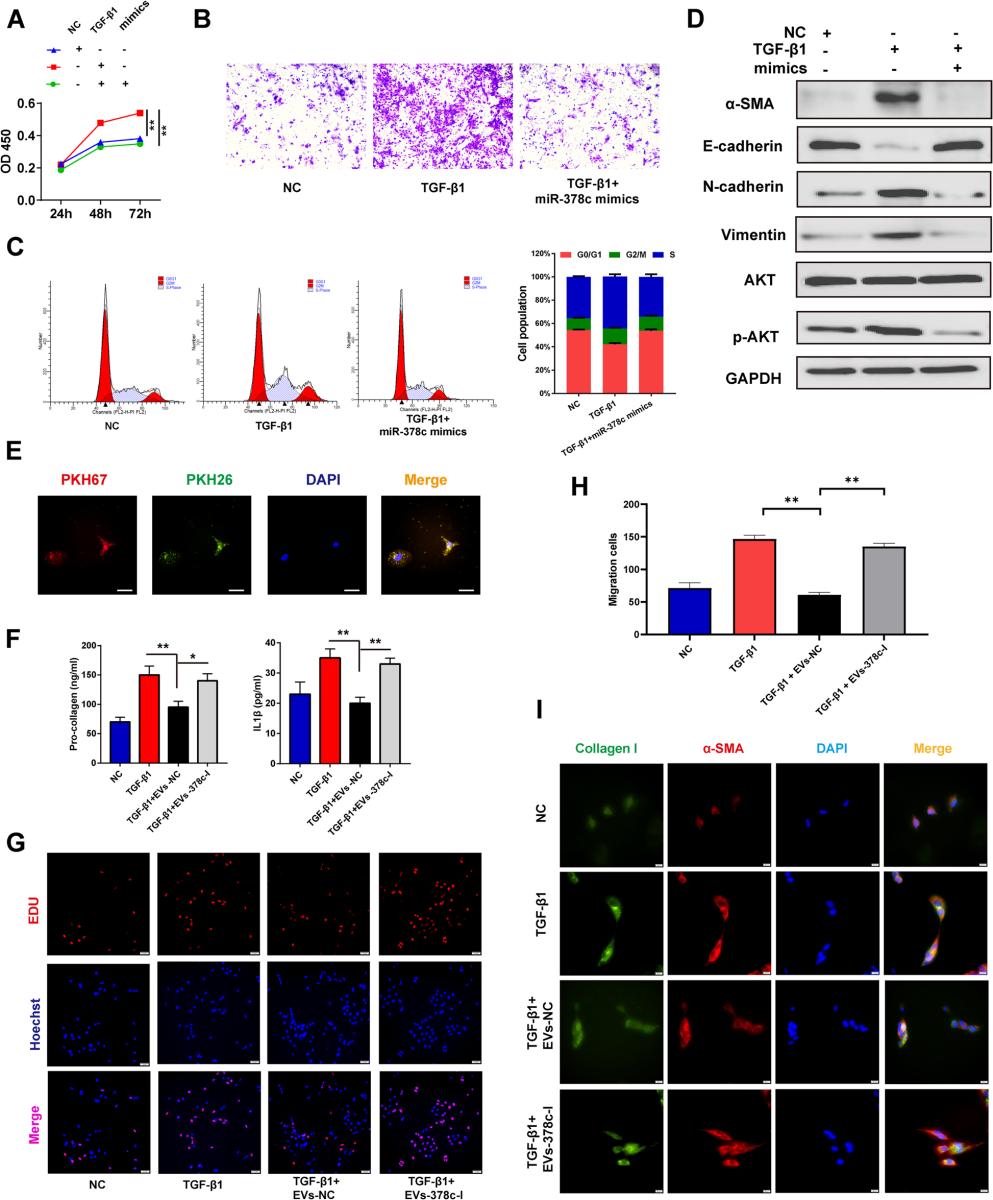
Pd-MSCs derived miR-378c inactivated HSCs. **A**–**C** CCK8, transwell, and flowcytometry assays were performed to detect the proliferation, migration, and cell cycle distribution of HSCs in each group. **D** Western blotting was used to detect the expression of fibrotic and EMT markers of HSCs at each group. **E** Uptake of extracellular vesicles by HSCs through PKH67 and PKH26 staining. **F**–**H** After different treatments, the secretion, proliferation, and migration activities of HSCs were detected by ELISA, EdU, and transwell assays, respectively. **I** The expression of fibrotic markers in HSCs at different groups was detected by immunofluorescence. TGF-β1, transformation growth factor- beta; α-SMA, α-smooth muscle actin. *P < 0.05; **P < 0.01

In parallel with the observations in 3D levels, CCK-8 showed that Pd-MSCs-EVs impeded the growth of HSCs stimulated by TGF-β1 treatment (Figure S4A). Flow cytometry analysis showed that simultaneous treatment of Pd-MSCs-EVs could clearly induce the cell cycle arrest in G1 phase compared with single treatment of TGF-β1 (Figure S4C), while neither TGF-β1 nor Pd-MSCs-EVs had significant effects on the apoptosis of HSCs (Figure S4B). Then, we evaluated the effects of miR-378c on HSCs. MiR-378c mimics significantly repressed proliferation and migration of HSCs, with a significant G0/G1 arrest in TGF-β1-activated HSCs (Fig. 7A–C). In addition, miR-378c obviously inhibited the expression of fibrosis and EMT markers in TGF-β1-activated HSCs (Fig. 7D). Consistently, Pd-MSCs-EVs-NC significantly alleviated the proliferation, secretion, and migration of HSCs activated by TGF-β1, whereas EVs-378c-I had no significant effects (Fig. 7E–H). Notably, immunofluorescence showed that Pd-MSCs-EVs could decrease the expression of α-SMA and Collagen I upregulated by TGF-β1, while extracellular vesicles with miR-378c-delepted showed no significant decreasing effects on the fibrotic markers (Fig. 7I). It suggested that EVs-derived miR-378c might alleviate fibrosis by inactivating HSCs.

### miR-378c alleviated fibrosis by inhibiting SKP2

**Fig. 8:**
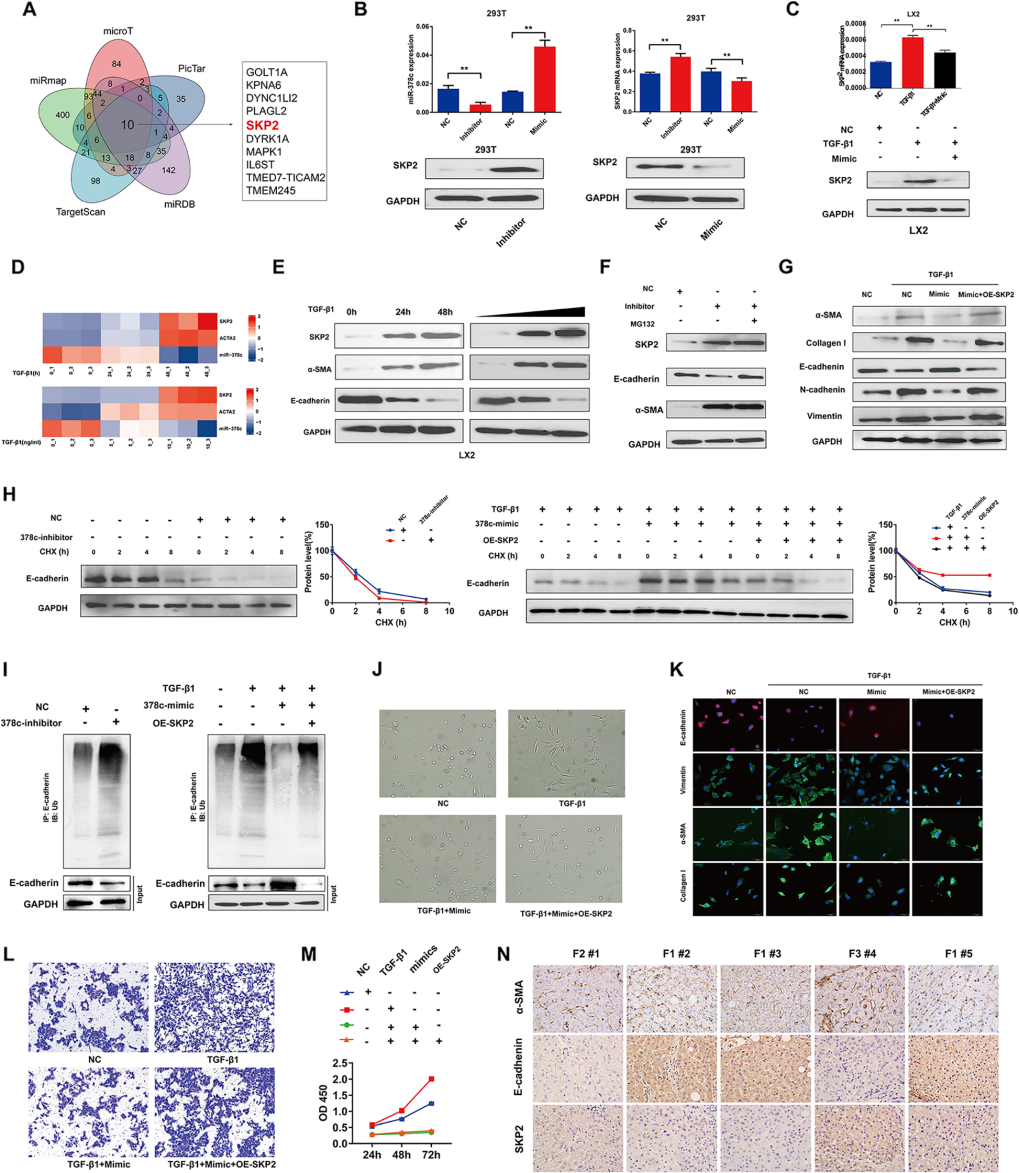
miR-378c alleviated liver fibrosis by targeting SKP2. **A** The potential targets of miR-378c were predicted by bioinformatic analyses. **B**, **C** The regulatory effects of miR-378c on SKP2 were validated in HEK-293 T and LX-2 cells. **D** The expression of SKP2, ACAT2, and miR-378c in LX-2 cells with TGF-β1 treatment at different doses and exposure time was detected by RT-qPCR. **E** The expression of SKP2, α-SMA, and E-cadherin in LX-2 cells with different treatments were detected by western blotting. **F**, **G** The expression of SKP2, α-SMA, and E-cadherin were detected by western blotting following different treatments. **H** The differences of E-cadherin protein degradation induced by CHX were detected in the various groups. **I** The differences in E-cadherin ubiquitination were detected in the groups with the treatments above. **J** The morphology of HSCs with TGF-β1, miR-378c mimics, and SKP2 overexpression. **K** The expression of fibrotic markers in HSCs at different groups was detected by immunofluorescence. **L**, **M** Transwell and CCK8 assays were performed to detect the migration and proliferation of HSCs in each group, respectively. **N** The SKP2, α-SMA, and E-cadherin expression in 5 human fibrotic tissues was detected by immunohistochemical staining. TGF-β1, transformation growth factor- beta; α-SMA, α-smooth muscle actin. **P < 0.01

Next, we explored the underlying mechanisms accounting for the anti-fibrotic effects of miR-378c. Following overlapping 5 bioinformatic datasets, 10 genes were predicted as the potential targets of miR-378c (Fig. 8A). Among them, S-Phase Kinase Associated Protein 2 (SKP2), correlated with fibrosis and the activation of HSCs, was chosen for further validation [26]. As expected, the mimics or inhibitor of miR-378c could significantly decrease or increase the SKP2 expression of 293 T cells at mRNA and protein levels (Fig. 8B). In addition, miR-378c mimics inhibited the upregulation of SKP2 induced by TGF-β1 in HSCs (Fig. 8C). Furthermore, as shown in Fig. 8D, E, TGF-β1 modulated the expression of SKP2, ACAT2(α-SMA), and miR-378c at dose- and time-dependent manner. Consistently, the fibrosis mouse model indicated that SKP2, E-cadherin, and α-SMA expression was obviously altered with fibrosis progression (Figure S5). The miR-378c inhibitor significantly upregulated expression of SKP2 and α-SMA with E-cadherin downregulation, whereas MG132 restored the alterations mediated by miR-378c inhibitor (Fig. 8F). As elucidated in Fig. 8G, miR-378c mimic remarkably modulated the expression of EMT and fibrosis markers of HSCs, which was further rescued by SKP2 overexpression. E-cadherin has been previously identified as a substrate of the E3 ubiquitin ligase SKP2. Accordingly, miR-378c inhibitor significantly accelerated the CHX-induced E-cadherin degradation and enhanced E-cadherin ubiquitination (Fig. 8H, I). In contrast, miR-378c stabilized E-cadherin protein with reduced ubiquitination, which was recovered by SKP2 overexpression. As shown in Fig. 8J, K, exogenous SKP2 rescued EMT morphology and the expression levels of EMT markers. Correspondingly, SKP2 also restored the proliferation and migration activities of HSCs that were inhibited by miR-378c mimics (Fig. 8L, M). As elucidated in the IHC staining of 5 clinically fibrosis tissues in Fig. 8N, SKP2 expression was positively correlated with α-SMA and negatively correlated with E-cadherin. The results above demonstrated that miR-378c might abate liver fibrosis by targeting SKP2 in HSCs.

## Discussion

Liver fibrosis is a chronic, progressive, and irreversible hepatic disease characterized by hepatocyte injury and structural remodeling of the tissue. In general, when confronted with an acute injury, the liver has the regenerative capacity to restore its original architecture and function in a relatively short time interval. However, when faced with etiologies causing chronic liver, the initiation of a series of inflammatory chronic wound healing responses significantly impairs its regenerative abilities [27]. As chronic liver injury accumulates and reaches an advanced stage, there is typically no therapeutic response to the general anti-inflammatory treatments currently available. Some anti-fibrotic agents, adenosine receptor antagonist-based solutions, and even MSC transplantation have shown some promise, but their efficacy is limited [28]. However, novel nanomedicine-based strategies can provide potential benefits for the prevention and treatment of liver fibrosis. Indeed, EVs from various types of MSCs have shown therapeutic effects on fibrotic diseases of various organs, including the heart, kidney, and lung [29]. Specifically in regard to liver fibrosis, the protective anti-fibrosis function of extracellular vesicles from UC-MSCs was observed in preclinical models [30, 31]. In contrast to other adult MSCs, Pd-MSCs have presented distinct advantages, including extensive abundant sources, lack of ethical concerns (donation at birth), low immunogenicity, effective immunomodulatory ability, high self-renewal and proliferative capacity, lower risk of virus infection, strong expansion capacity, and less adverse effects on the donor [32–34]. However, there are relatively less studies focusing on the therapeutic roles of secreted production from Pd-MSCs. In the current study, to better mimic the microenvironment of the fibrotic liver, the effects of Pd-MSCs-EVs were evaluated in liver organoids and spheroids.

Following our previous work, we initially constructed a liver organoid model using multiple-cell types involved in liver tissues (hepatocytes, Kupffer cells, liver sinusoidal endothelial cells, and HSCs). We observed the organoids for about 6 weeks and noted the multi-cellular self-assembled liver organoids maintained their structural integrity and viability status during the initial weeks post-seeding. Once the liver organoids were established in culture, they were subjected to fibrosis induction. In contrast to the CCL4-induced liver fibrotic model in vivo, lipopolysaccharide (LPS), allyl alcohol, and TGF-β1 were commonly adopted for hepatic fibrosis in vitro [35]. Thus, based on the dose-dependent evaluation, TGF-β1 was administrated at an optimal concentration to induce fibrosis in liver organoids. Subsequently, TGF-β1 treatment led to the decline in liver organoid viability and elevated expression of fibrotic markers and inflammatory factors, suggesting that the liver organoids in normal status might have been partially transferred to fibrotic liver organoids. Based on this fibrotic phenotype, our study continued to explore the effects of extracellular vesicles from Pd-MSCs on liver fibrosis in the organoids model.

Although extracellular vesicles were evaluated in various 2D culture models, little evidence has been found associating the optimal concentration with uptake by organoids. In general, for the normal 2D culture system, the common time point for uptake observation is 4–6 h. However, based on the comparison between 6 and 24 h, the best time point of uptake by liver organoids was instead found to be at 24 h. The discrepancy might be attributed to the complex cellular components and compact ECM components surrounding the cells. As expected, further administration of Pd-MSCs-EVs at 40 μg/ml significantly recovered the albumin secretion, while the observed difference in the growth status was not significant. Notably, Pd-MSCs derived extracellular vesicles also suppressed TGF-β1-induced secretion of inflammatory factors and expression of fibrotic markers. It indicated that extracellular vesicles derived from MSCs showed promising capacity of alleviating fibrosis, which corroborated the findings of a great deal of the previous in vivo work recommending MSCs-EVs as a promising anti-fibrosis medicine [15].

EV-derived miRNAs might be crucial components that govern exosome activities. Thus, we further identified the key component in Pd-MSC-derived extracellular vesicles. Following omics investigations and validations in liver organoids, miR-378c was chosen as potential candidate. Further depletion of miR-378c significantly decreased the therapeutic efficacy of extracellular vesicles in TGF-β1-administrated liver organoids, suggesting miR-378c might account for the anti-fibrosis effects of Pd-MSC-derived extracellular vesicles. Previous studies have highlighted the negative regulation of miR-378c on tumorigenesis, which is characterized by inhibiting the malignant behaviors including proliferation, migration, and EMT phenotypes [36]. Though there is no evidence suggesting the correlation of miR-378c with liver fibrosis, the current study proposed it as a potential anti-fibrosis component in Pd-MSC-derived extracellular vesicles.

It is evident that HSCs are mainly responsible for both fibrogenesis and fibrolysis of the liver [37]. Activation of HSC has been widely recognized as the central event of liver fibrosis [38]. Previous studies showed that the CM of MSCs promoted apoptosis and inhibited proliferation of HSC line LX-2 [39]. Furthermore, amnion-derived MSCs (AMSCs) significantly inhibited HSCs activation in vitro and in vivo [40]. Similarly, in this study, treatment of Pd-MSCs-EVs could inhibit the proliferation, the migration and induce cell cycle arrest of HSCs. Then, the effects of Pd-MSCs-EVs were evaluated on 3D HSC-derived spheroids. Interestingly, the proliferation, metabolism activity, and expression of fibrotic markers of HSC-derived spheroids were also decreased when treated with Pd-MSCs-EVs. Consistent with the observations in liver organoids, miR-378c mimics could decrease the activity of HSCs. In addition, miR-378c-delepted extracellular vesicles also lose the inhibitory effects on HSCs, suggesting the EV-derived miR-378c might be the key factor against fibrosis in Pd-MSC-derived extracellular vesicles. Then SKP2 was predicted as the potential downstream target of miR-378c by using bioinformatic prediction and molecular validation. Previous studies demonstrated that SKP2, as a E3 ubiquitin ligase, was associated with fibrosis and involved in TGF-β1-induced EMT and E-cadherin ubiquitination [41, 42]. Then we further investigated the effects of miR-378c/SKP2 axis on E-cadherin stabilization. As expected, miR-378c stabilized E-cadherin with reduced ubiquitination, whereas SKP2 overexpression accelerated E-cadherin degradation and elevated ubiquitination levels. In addition, exogenous SKP2 could rescue the fibrotic and EMT markers of HSCs repressed by miR-378c, suggesting that miR-378c might abate liver fibrosis by targeting SKP2 in HSCs.

**Fig. 9:**
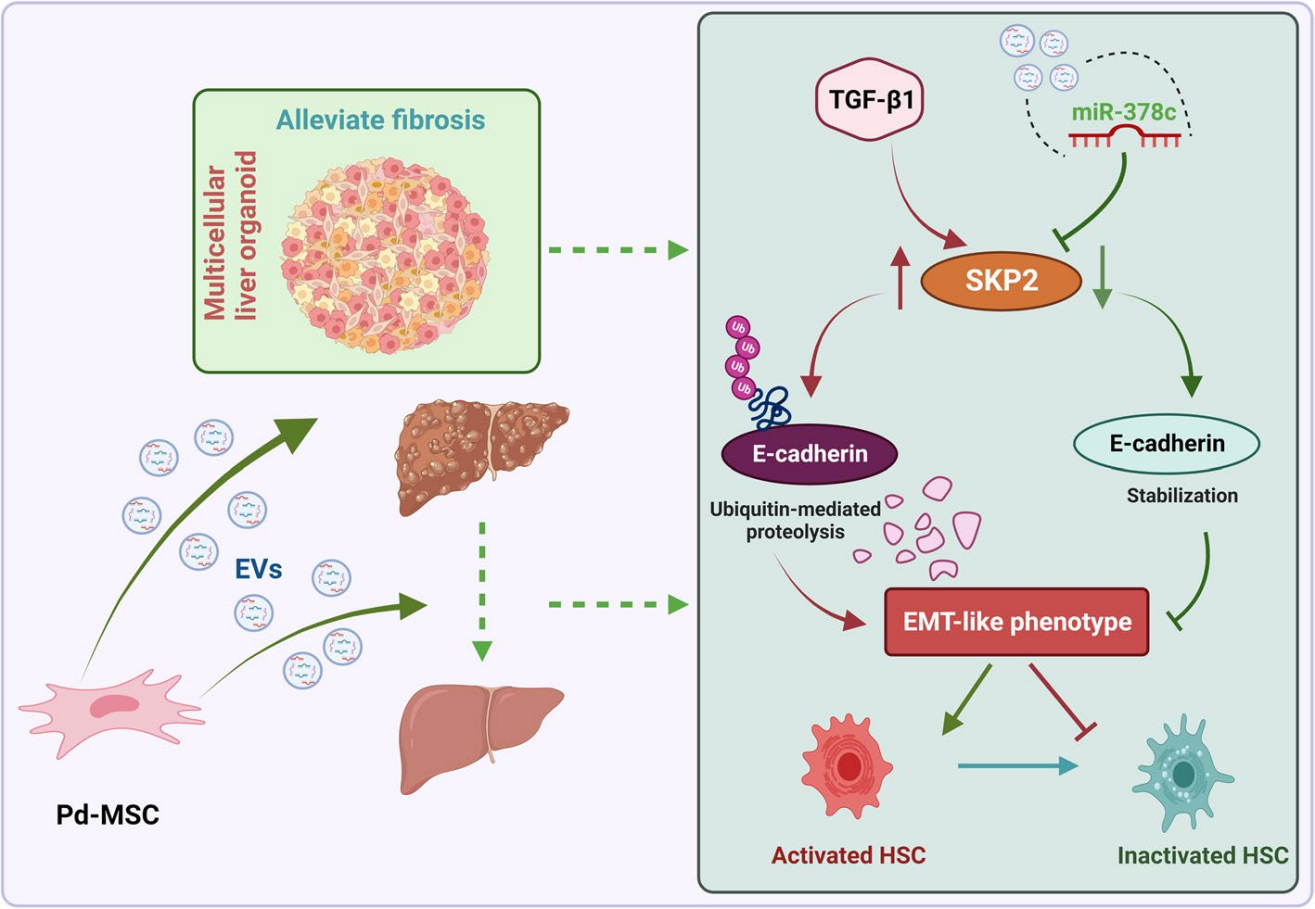
The mechanism graph of this study. Based on the observation and validations in organoid, 2D HSC model, and liver tissues, Pd-MSCs derived extracellular vesicles might alleviate liver fibrosis by inactivating HSCs in a miR-378c/SKP2 axis

It is well known that there are three EMT subtypes. Type 1 and type 2 are implicated in embryogenesis and fibrogenesis during chronic tissue damage, while type 3 contributes to the tumor metastasis and invasion [40]. Historically, it has commonly been assumed that EMT is involved somehow in tumor metastasis and chemoresistance [43]. However, recent studies have shown that EMT has a strong, contributory impact on the development of fibrosis [44]. Specifically focusing on liver, EMT has been considered as a contributory factor during liver fibrogenesis in rats or mice models [45, 46]. In the whole liver organoid, Pd-MSC-derived extracellular vesicles could inhibit the EMT markers, while miR-378c-delepted extracellular vesicles had less inhibitory effects. A previous study demonstrated that hepatocytes EMT had less correlations with liver fibrosis [47]. Then, we focused on the fibrosis-related HSC. Classically, EMT process plays a pivotal role in the activation of HSC, which facilitates the morphological transition to myofibroblast [48–50]. Likewise, previous studies indicated that pro-EMT factors could induce HSC trans-differentiation and liver fibrosis [51]. In the current study, extracellular vesicles and miR-378c led to a significant decline in these EMT markers of HSCs. However, miR-378c-delepted extracellular vesicles or SKP2 overexpression rescued the EMT phenotypes of HCSs. Hence, based on the assays above, it could be conceivably hypothesized that anti-fibrosis effects of Pd-MSCs derived extracellular vesicles might be partially attributed to impeding EMT process of HSCs in a miR-378c/SKP2-dependent manner (Fig. 9).

## Conclusions

In summary, anti-fibrotic and anti-inflammatory effects were identified in liver organoids with the administration of Pd-MSC-derived extracellular vesicles. The therapeutic effects of these extracellular vesicles might be attributed to the inactivation of HSCs, characterized by the reduction of ECM and reversal of EMT in HSCs mediated by EVs-derived miR-378c. Therefore, this cell-free therapy could be considered as an alternative, yet still efficacious approach to the treatment of fibrotic liver disease. Additionally, TGF-β1-induced liver fibrotic organoid model could be a promising option for anti-fibrosis drug screening.

### Supplementary Information

**Additional file 1: Figure S1.** The viability of liver organoids treated with TGF-β1. **Figure S2.** Pd-MSCs-EVs reversed TGF-β1-induced activation of HSCs in a spheroid model. **Figure S3.** Fibrotic markers in HSCs treated with TGF-β1 and Pd-MSCs-EVs. **Figure S4.** The effects of Pd-MSCs-EVs on apoptosis and cell cycle of HSCs. **Figure S5.** SKP2/E-cadherin axis in CCL4-induced fibrosis model. **Table S1.** The primer sequences.

**Additional file 2.**

## Data Availability

The data that support the findings of this study are available from the corresponding author upon reasonable request.

## Abbreviations

MSCs: Mesenchymal stem/stromal cells
EVs: Extracellular vesicles
HSCs: Hepatic stellate cells
TGF-β1: Transformation growth factor- beta 1
EMT: Epithelial-mesenchymal transition
SKP2: S-Phase Kinase Associated Protein 2
HCC: Hepatocellular carcinoma
ECM: Extracellular cell matrix
TBST: Tris-buffered saline tween
DAB: 3,3′-Diaminobenzidine
IL-1β: Interleukin-1 β
SD: Standard deviation
