# Supplementary material for: Placenta mesenchymal stem cell-derived extracellular vesicles alleviate liver fibrosis by inactivating hepatic stellate cells through a miR-378c/SKP2 axis
Source: Inflamm Regen. 2023 Oct 5;43:47. doi: 10.1186/s41232-023-00297-z (PMC10557276; doi:10.1186/s41232-023-00297-z)
Supplement: Supplementary file 1 — Additional file 1: Figure S1. The viability of liver organoids treated with TGF-β1. Figure S2. Pd-MSCs-EVs reversed TGF-β1-induced activation of HSCs in a spheroid model. Figure S3. Fibrotic markers in HSCs treated with TGF-β1 and Pd-MSCs-EVs. Figure S4. The effects of Pd-MSCs-EVs on apoptosis and cell cycle of HSCs. Figure S5. SKP2/E-cadherin axis in CCL4-induced fibrosis model. Table S1. The primer sequences. [file 41232_2023_297_MOESM1_ESM.docx]

**
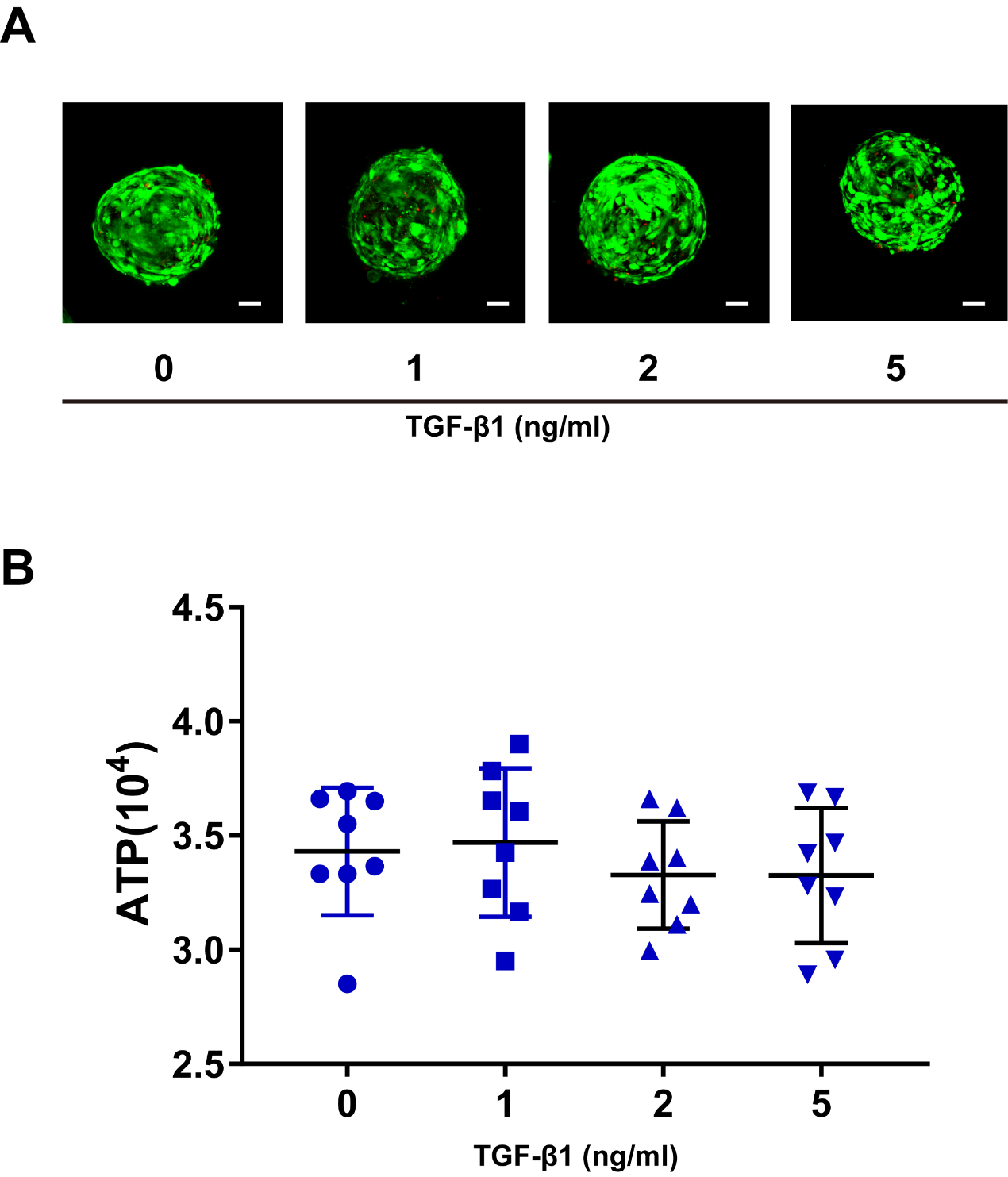
Figure S1. The viability of liver organoids treated with TGF-β1.**

**A,** Live/Dead staining was performed in liver organoids treated with TGF-β at different concentrations. **B,** The growth status of liver organoids treated with TGF-β at different concentrations was detected by 3D viability assay. TGF-β, transformation growth factor- beta. Bar scale, 50μm.


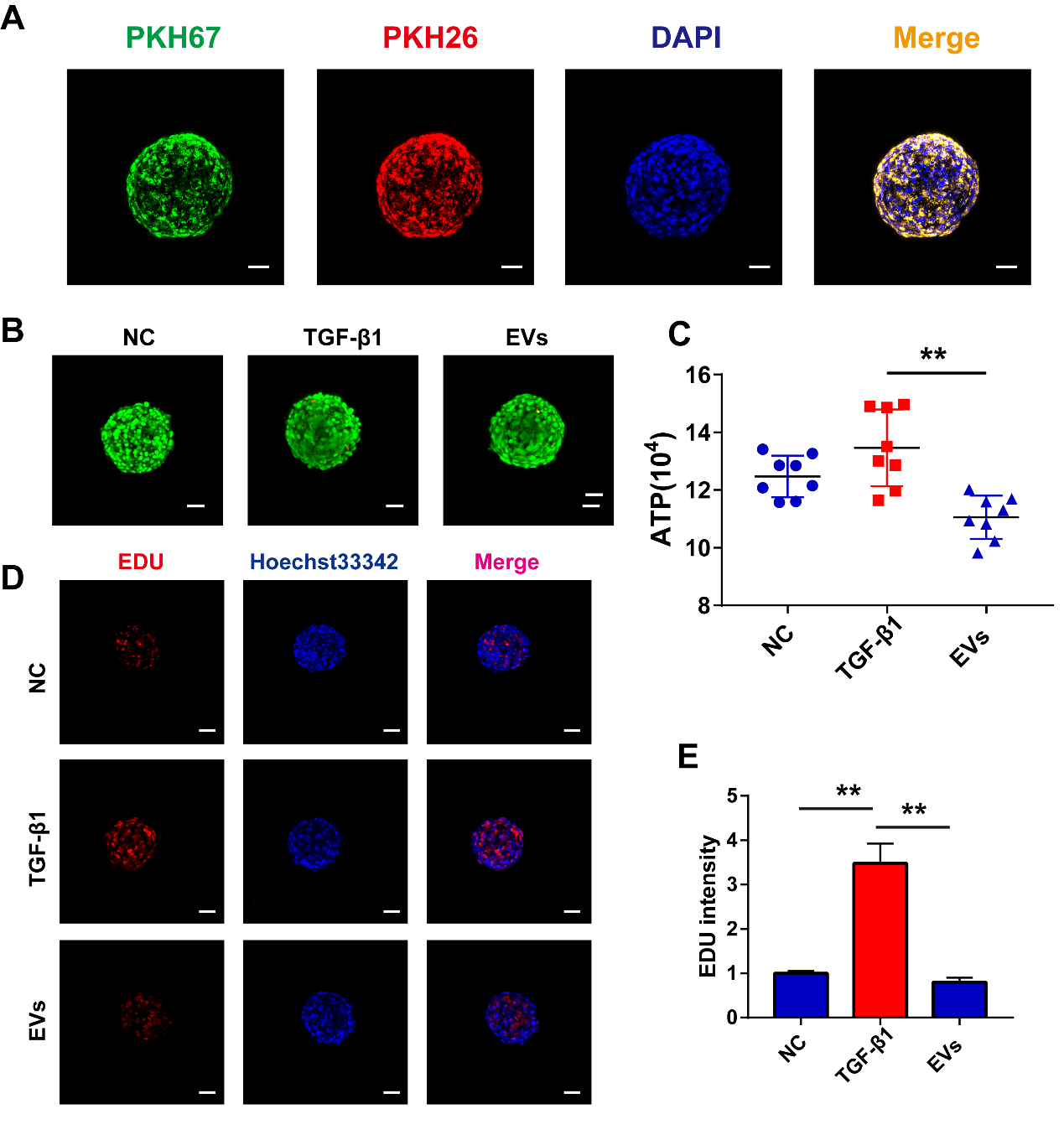


**Figure S2. Pd-MSCs-EVs reversed TGF-β1-induced activation of HSCs in a spheroid model.**

**A**, The uptake of EVs by HSCs-derived spheroids through PKH67 and PKH26 staining. **B,** The HSCs-derived spheroids were treated with TGF-β1 and Pd-MSCs-EVs and then detected for growth status by Live/Dead staining assay. **C,** The growth status of HSCs-derived spheroids was detected by 3D viability assay. **D&E,** The proliferation of HSCs-derived spheroids with TGF-β1 and Pd-MSCs-EVs was analyzed by EdU assay. TGF-β, transformation growth factor- beta 1; HSCs, hepatic stellate cells; α-SMA, α-smooth muscle actin; EdU, 5-Ethynyl-2’- deoxyuridine. Bar scale, 50μm. *, *P*<0.05; **, *P*<0.01.


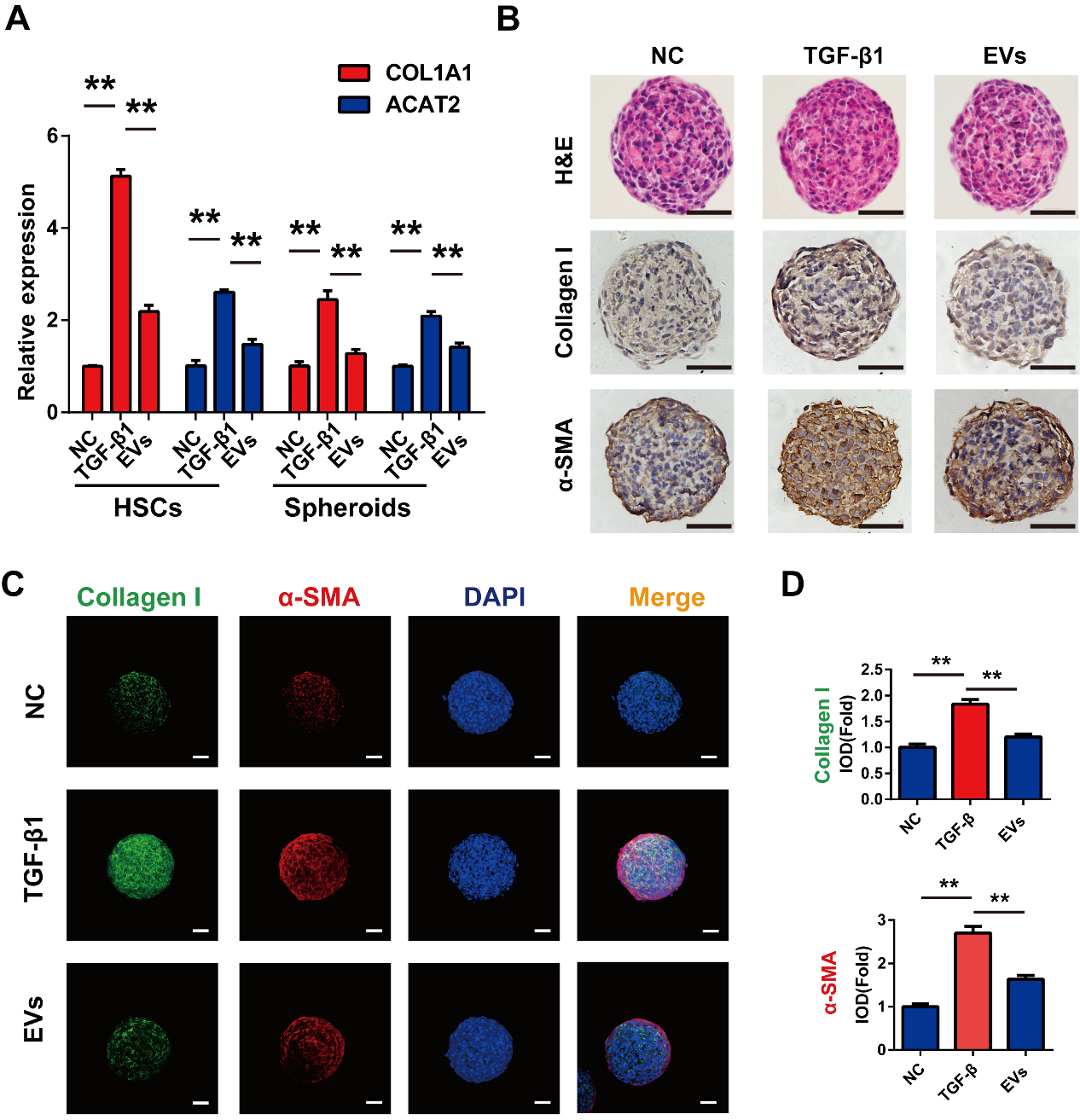


**Figure S3. Fibrotic markers in HSCs treated with TGF-β1 and Pd-MSCs-EVs**

**A,** The mRNA expression of COL1A1 and ACAT2 in HSCs and HSCs-derived spheroids detected by RT-qPCR. **B,** The H&E staining and IHC staining (collagen I and α-SMA) of the HSCs-derived spheroids treated with TGF-β1 and Pd-MSCs-EVs. **C-E,** The expression of collagen I and α-SMA was detected in HSCs-derived spheroids treated with TGF-β1 and Pd-MSCs-EVs by immunofluorescence. TGF-β1, transformation growth factor- beta 1; EVs, extracellular vesicles; α-SMA, α-smooth muscle actin. Bar scale, 50μm. *, *P*<0.05; **, *P*<0.01.


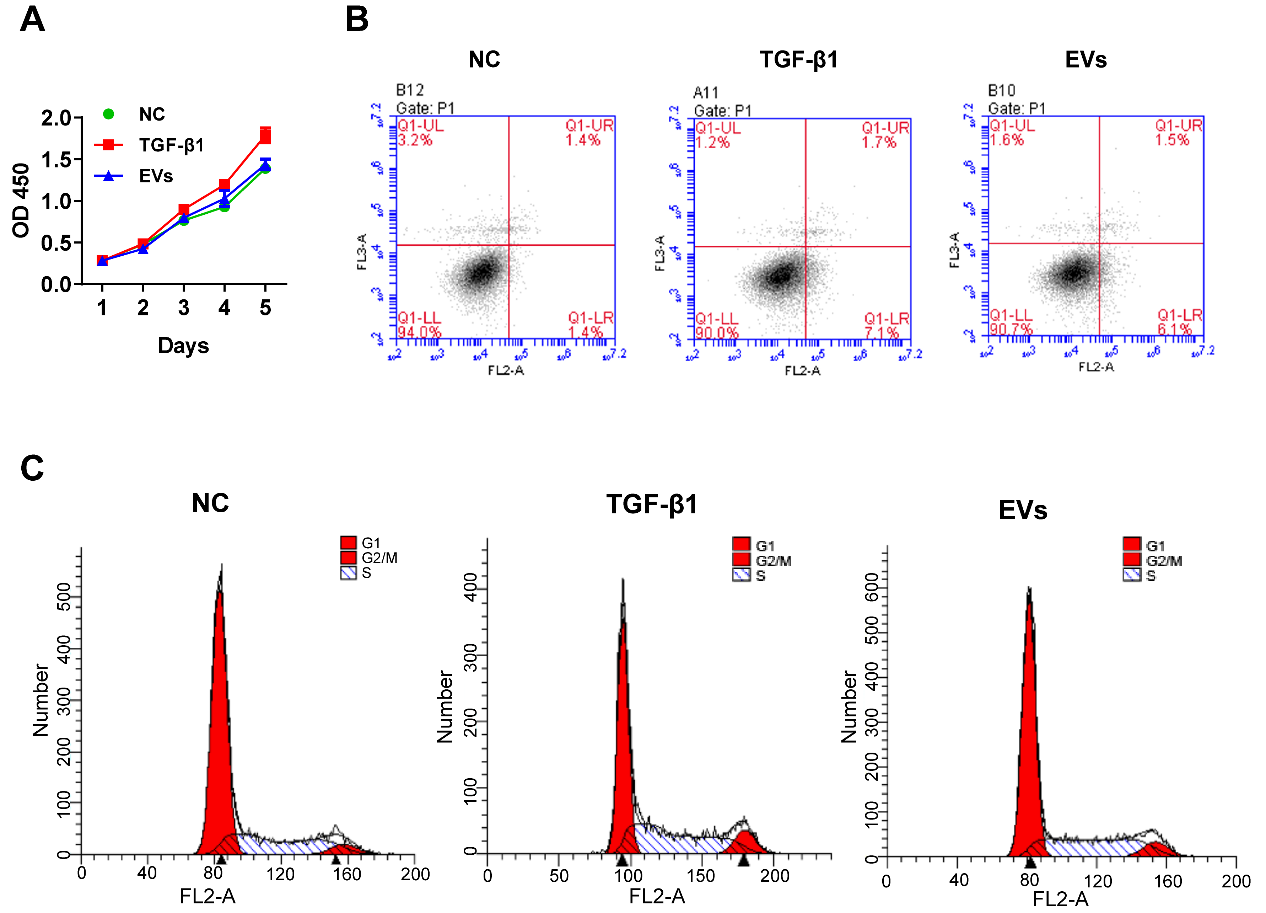


**Figure S4. The effects of Pd-MSCs-EVs on apoptosis and cell cycle of HSCs**

**A,** CCK8 was performed to detect the proliferation of HSCs. **B,** Flow cytometry was performed to detect the apoptosis in HSCs treated with TGF-β1 and Pd-MSCs-EVs. **C,** Cell cycle of HSCs treated with TGF-β1 and Pd-MSCs-EVs was detected by flow cytometry.


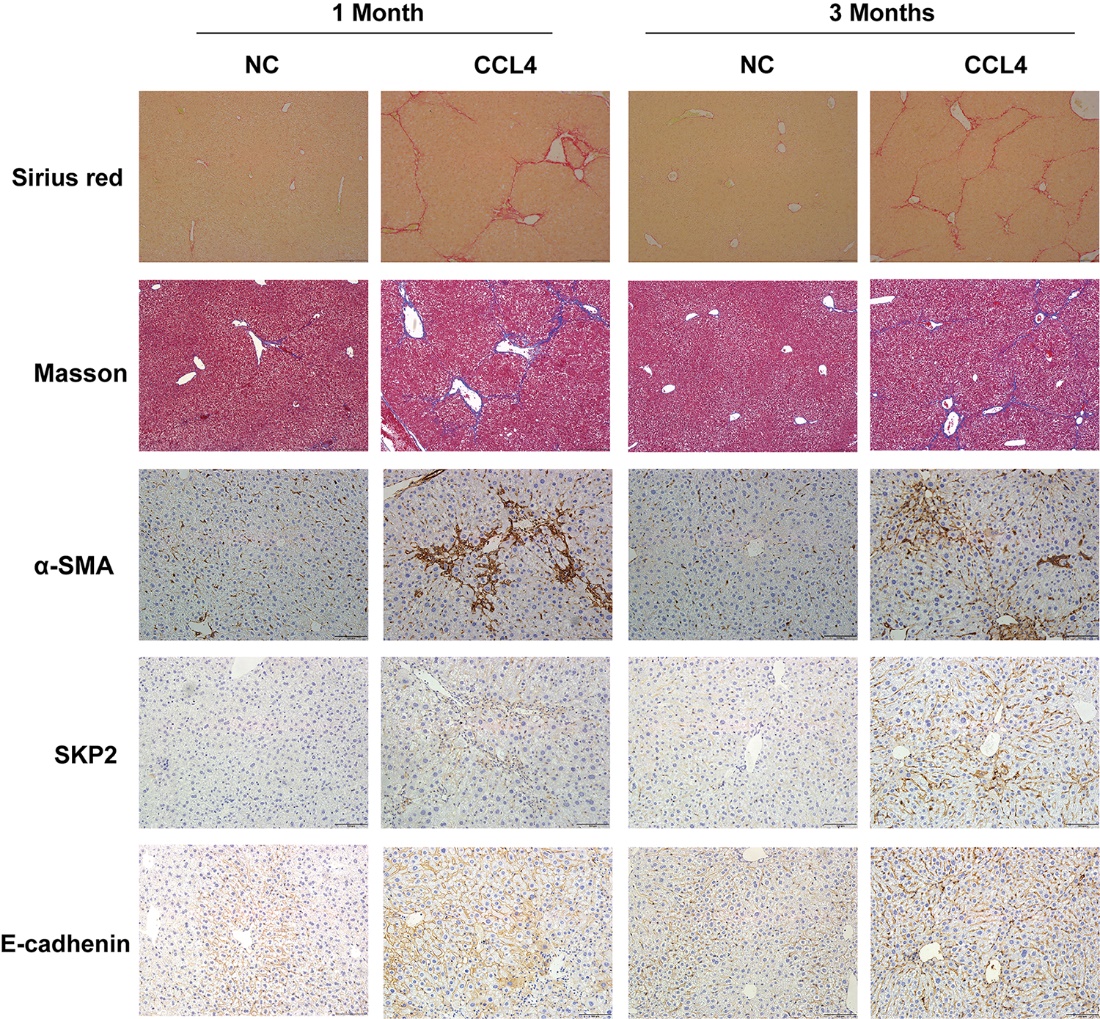


**Figure S5. SKP2/E-cadherin axis in CCL4-induced fibrosis model**

The CCL4-induced liver fibrosis model was validated by Sirius red staining and Masson staining at the time period of 1^st^ month and 3^rd^ month. The expression of α-SMA, SKP2, and E-cadherin in the liver tissues at different groups was detected by IHC staining. α-SMA, α-smooth muscle actin.

**Table S1. The primer sequences.**

| **Gene** | **Sequence (5’~3’)** |
| --- | --- |
| **ACTA2** | F: AAAAGACAGCTACGTGGGTGA  R: GCCATGTTCTATCGGGTACTTC |
| **ALB** | F: TGCAACTCTTCGTGAAACCTATG  R: ACATCAACCTCTGGTCTCACC |
| **COL1A1** | F: GAGGGCCAAGACGAAGACATC  R: CAGATCACGTCATCGCACAAC |
| **COL1A2** | F: GTTGCTGCTTGCAGTAACCTT  R: AGGGCCAAGTCCAACTCCTT |
| **GAPDH** | F: GGAGCGAGATCCCTCCAAAAT  R: GGCTGTTGTCATACTTCTCATGG |
| **CDH1** | F: CGAGAGCTACACGTTCACGG  R: GGGTGTCGAGGGAAAAATAGG |
| **VIM** | F: GACGCCATCAACACCGAGTT  R: CTTTGTCGTTGGTTAGCTGGT |
| **TWIST1** | F: GTCCGCAGTCTTACGAGGAG  R: GCTTGAGGGTCTGAATCTTGCT |
| **SNAI1** | F: TCGGAAGCCTAACTACAGCGA  R: AGATGAGCATTGGCAGCGAG |
| **CDH2** | F: TCAGGCGTCTGTAGAGGCTT  R: ATGCACATCCTTCGATAAGACTG |
| **FN1** | F: CGGTGGCTGTCAGTCAAAG  R: AAACCTCGGCTTCCTCCATAA |
