# Supplementary material for: Placenta mesenchymal stem cell-derived extracellular vesicles alleviate liver fibrosis by inactivating hepatic stellate cells through a miR-378c/SKP2 axis
Source: Inflamm Regen. 2023 Oct 5;43:47. doi: 10.1186/s41232-023-00297-z (PMC10557276; doi:10.1186/s41232-023-00297-z)

Figure 3E

CD81

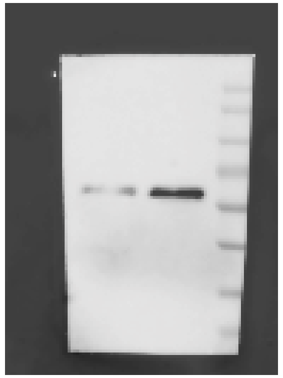

CD9

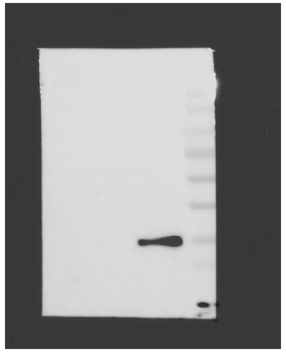

TSG101

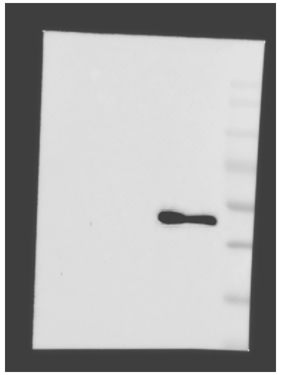

CD63

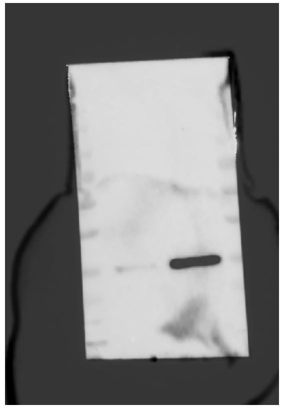

Figure 7D

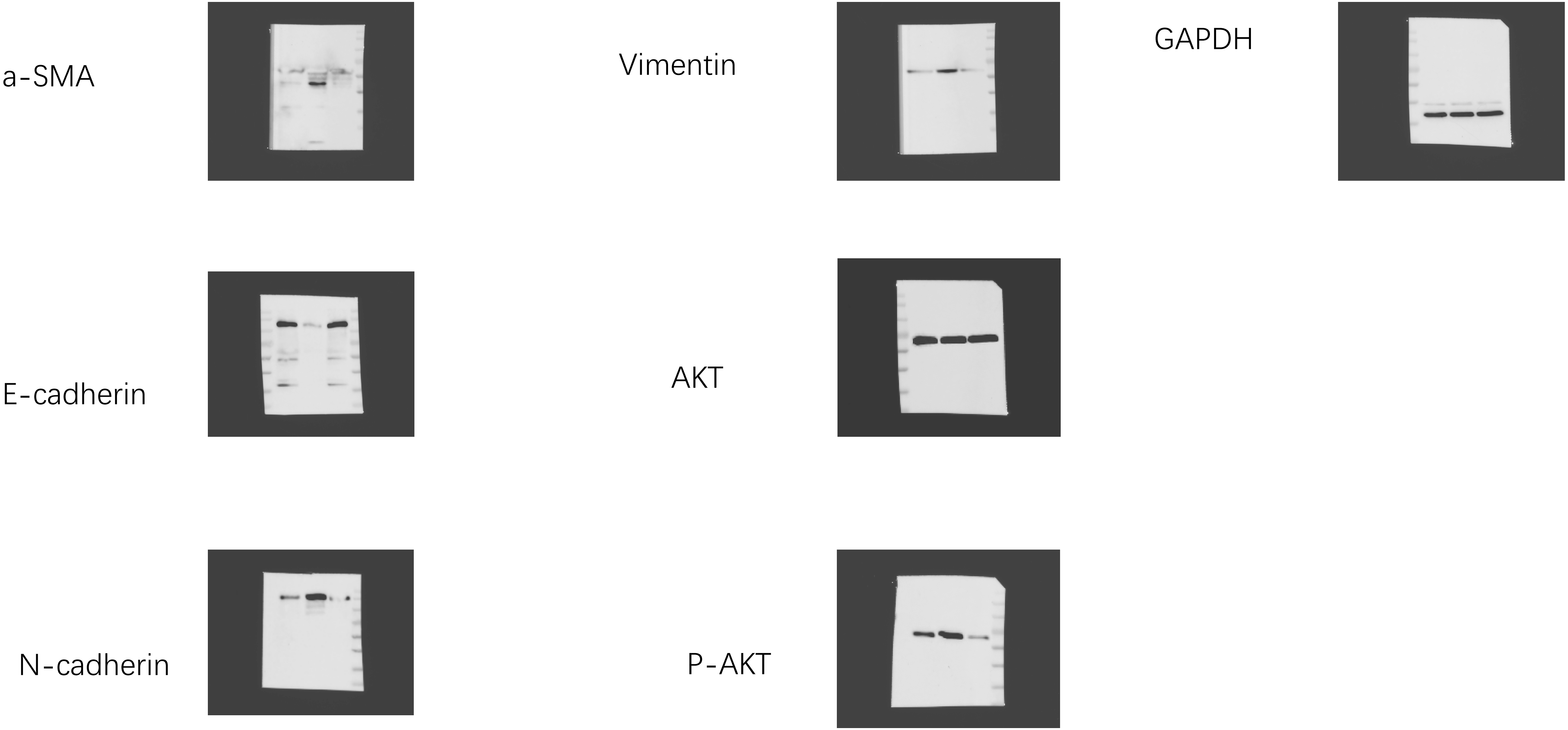

Figure 8B

SKP2

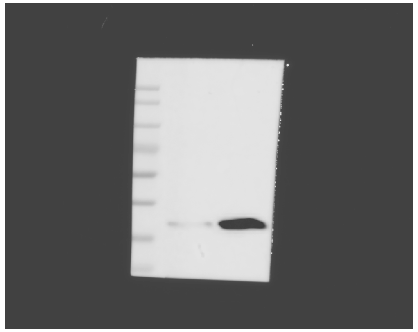

SKP2

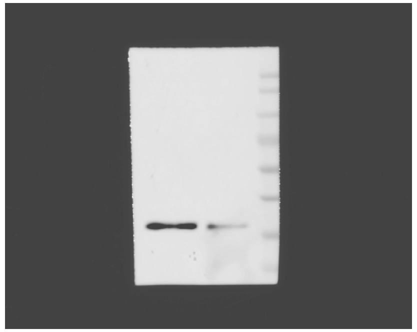

GAPDH

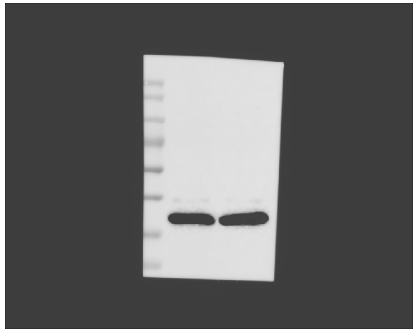

GAPDH

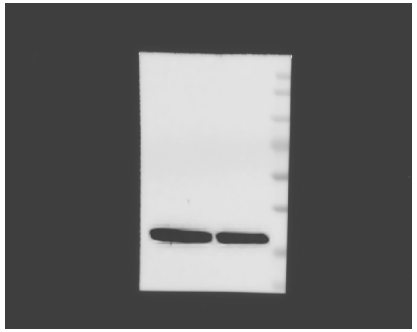

Figure 8C

SKP2

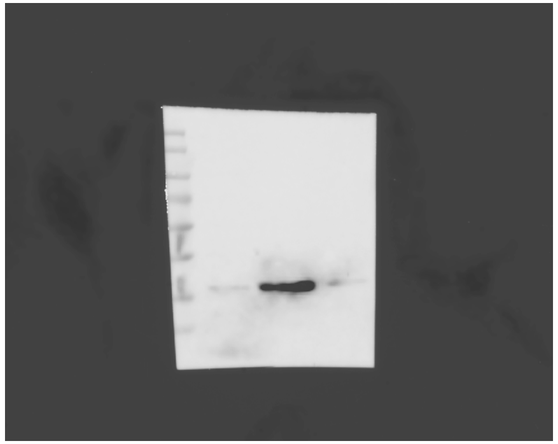

GAPDH

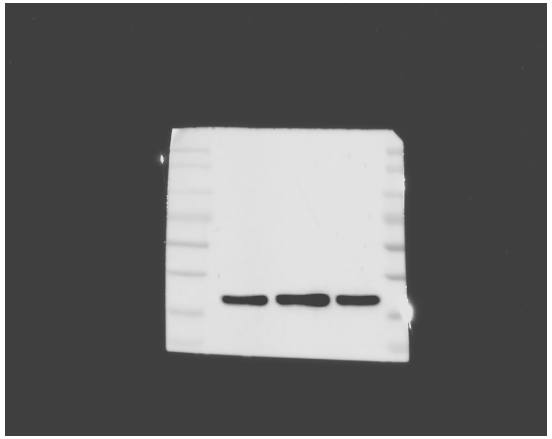

Figure 8E

Right panel

SKP2

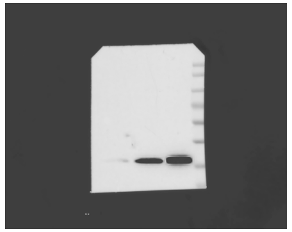

a-SMA

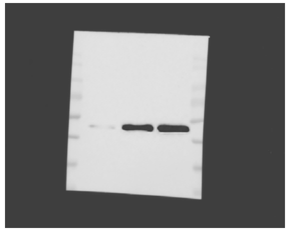

E-cadherin

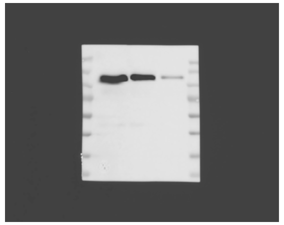

GAPDH

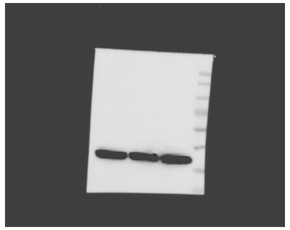

Left panel

SKP2

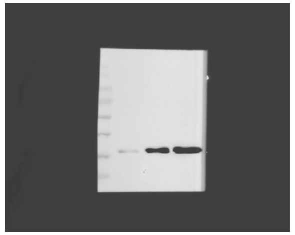

a-SMA

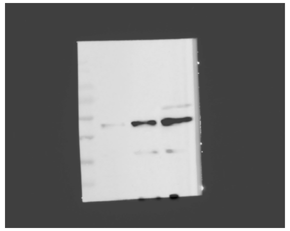

E-cadherin

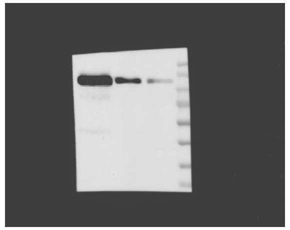

GAPDH

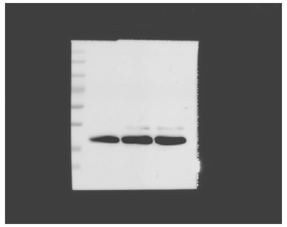

Figure 8F

SKP2

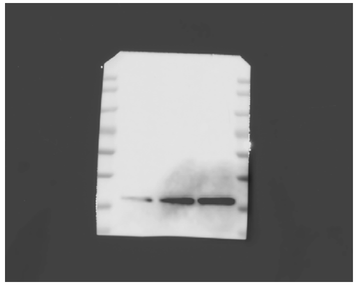

a-SMA

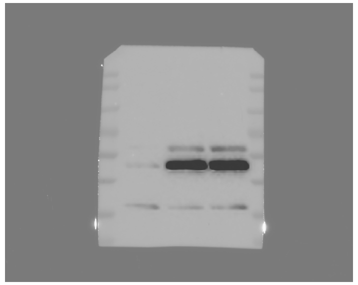

E-cadherin

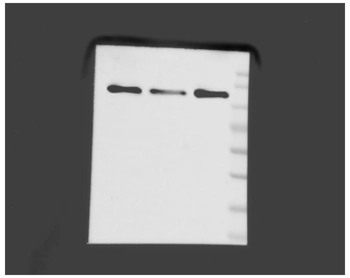

GAPDH

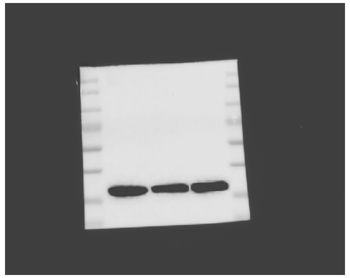

Figure 8G

E-cadherin

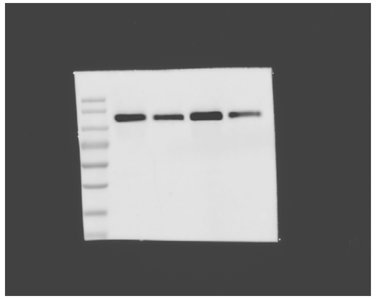

a-SMA

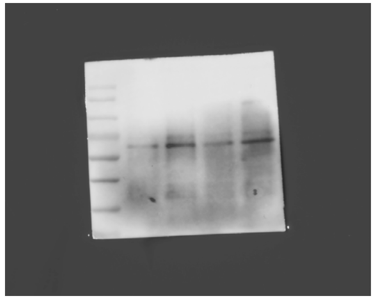

GAPDH

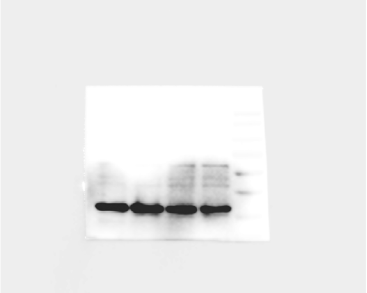

N-cadherin

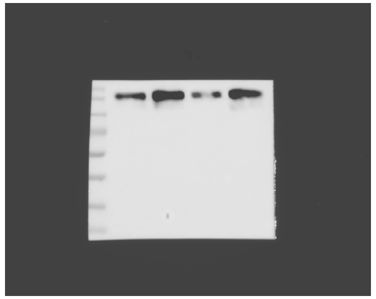

Collagen I

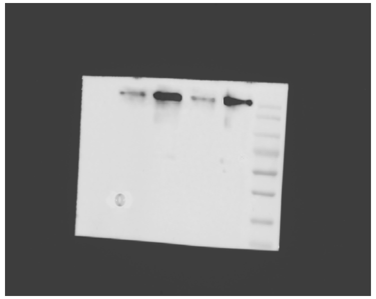

Vimentin

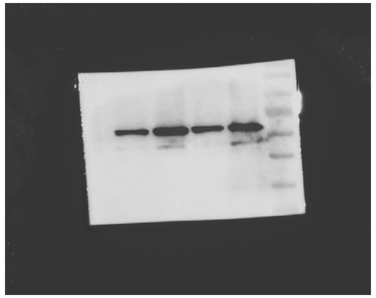

Figure 8H

E-cadherin

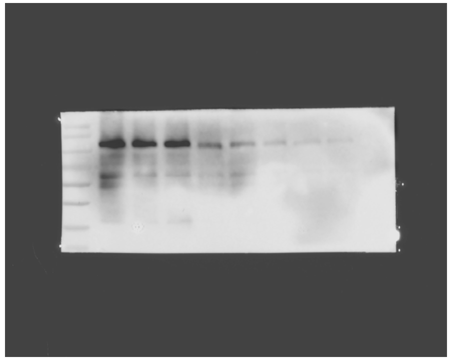

E-cadherin

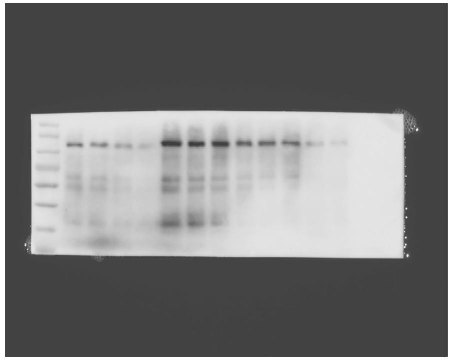

GAPDH

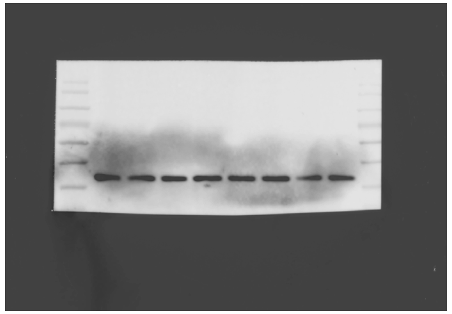

GAPDH

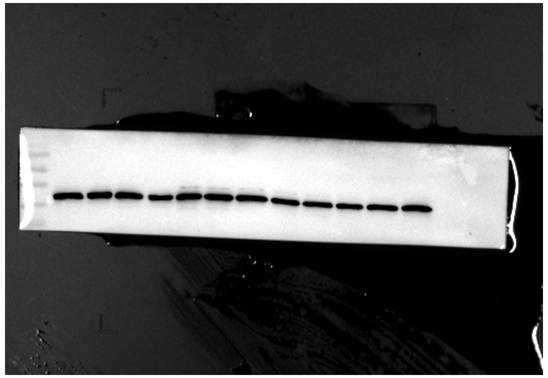

Figure 8I

IP:E-cadherin
IB:Ub

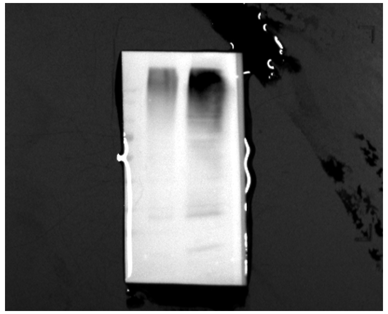

IP:E-cadherin
IB:Ub

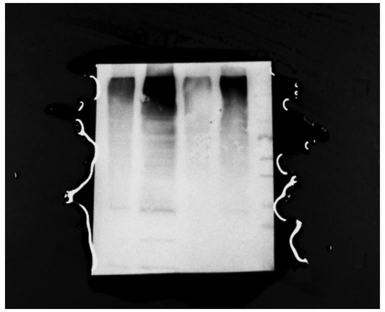

E-cadherin

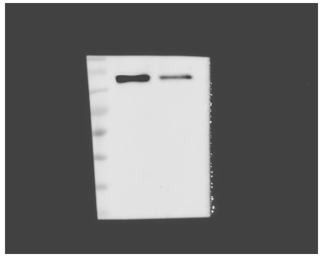

E-cadherin

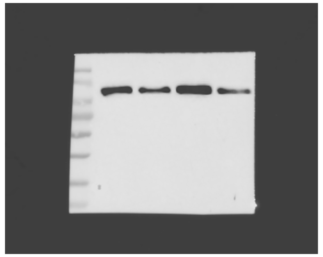

GAPDH

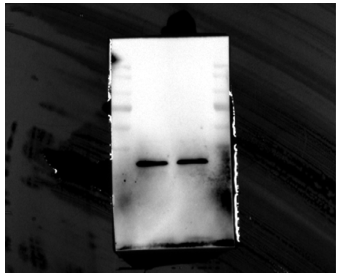

GAPDH

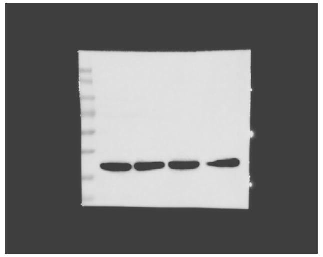

Supplement: Supplementary file 2 — Additional file 2. [file 41232_2023_297_MOESM2_ESM.pdf]
